# Defining the genetic determinants of CD8^+^ T cell receptor repertoire in the context of immune checkpoint blockade

**DOI:** 10.1126/sciadv.adu3461

**Published:** 2025-07-25

**Authors:** Esther S. Ng, Gusztav Milotay, Orion Tong, Chelsea A. Taylor, Shawn Sun, Guangyi Niu, Robert Watson, Bo Sun, Sophie MacKay, James J. Gilchrist, Martin Little, Benjamin P. Fairfax, Yang Luo

**Affiliations:** ^1^ Kennedy Institute of Rheumatology, Oxford, UK.; ^2^ MRC Weatherall Institute of Molecular Medicine, University of Oxford, Oxford, UK.; ^3^ Department of Oncology, University of Oxford, Oxford, UK.; ^4^ Cancer and Haematology Centre, Oxford University Hospitals NHS Foundation Trust, Oxford, UK.; ^5^ Nuffield Department of Clinical Neuroscience, University of Oxford, Oxford, UK.; ^6^ Department of Paediatrics, University of Oxford, Oxford, UK.; ^7^ NIHR Oxford Biomedical Research Centre, Oxford, UK.

## Abstract

The relationship between genetic variation and CD8^+^ T cell receptor (TCR) repertoire usage in patients receiving immune checkpoint blockade (ICB) therapy for cancer is unexplored. We have conducted a genome-wide and human leukocyte antigen (HLA)–focused analysis of CD8^+^ TCR repertoire to identify genetic determinants of variable gene (V-gene) and CDR3 *K*-nucleotide oligomer usage from samples taken before and after ICB (*n* = 250). We identify 11 cis and 10 trans V-gene associations, primarily to the MHC, that meet genome-wide significance. TCR clones containing HLA associated V-genes were less stable across treatment, while, at the single-cell level, genetically associated clones demonstrate subset enrichment and increased tumor reactivity expression profiles. Notably, patients with HLA-matched TCR clones demonstrate improved overall survival. Our work indicates a complex relationship between genotype and TCR repertoire in the context of ICB treatment, with implications for understanding factors relating to therapeutic response and patient outcomes.

## Teaser

Immune checkpoint blockade influences the genetically determined relationship between TCR repertoire and HLA alleles in cancer.

## INTRODUCTION

Immune checkpoint proteins, of which the cell-surface receptors programmed cell death protein 1 (PD-1) and cytotoxic T lymphocyte–associated antigen 4 (CTLA-4) are archetypal examples, play key roles in negatively regulating T cell responses, limiting deleterious inflammation and autoimmunity. In the context of cancer, induction and ligation of PD-1 and CTLA-4 are implicated in the development of T cell exhaustion and suppression of antitumor immunity. Correspondingly, treatment with immune checkpoint blockade (ICB) leads to reinvigoration of exhausted T cells, increasing their proliferation and cytotoxicity and broadening the T cell receptor (TCR) repertoire (*1*–*3*). Treatment with ICB has improved clinical outcomes across numerous cancers, most notably metastatic melanoma (MM) where treatment with combination ICB to CTLA-4 (ipilimumab) and PD-1 (nivolumab) for MM is associated with extending median survival from months to beyond 5 years (*4*, *5*).

CD8^+^ T cells are central to T cell–mediated antitumor responses to both neoantigens and tumor-associated antigens presented by class I human leukocyte antigen (HLA) alleles in complex with β-2 microglobulin. Successful recognition of HLA-presented antigens uses TCR incorporating α and β chains encoded on chromosome 14 and 9, respectively. Within the TCRβ chain, the first and second complementarity-determining regions (CDR1 and CDR2) primarily interface with HLA α helices, while interactions with the hypervariable CDR3 region are the critical determinant of peptide recognition (*6*). As such, in both the α and β chains, the CDR3 loops have the highest sequence diversity and are the principal determinants of receptor specificity (*7*). The CDR1 and CDR2 loops are determined by the variable gene (V-gene) segment in the TCR, while CDR3 hypervariability is secondary to V(D)J recombination during early T cell maturation. The process of gene segment recombination to encode a final full-length transcript results in a combinatorial possibility of 10^10^ possible TCR sequences (*8*), vital for enabling recognition of a vast array of self or pathogen derived peptides.

At the individual level, the TCR repertoire is determined by a complex interplay between antigen exposure and HLA determined propensity to present specific peptides. Early thymic selection is crucial in repertoire determination, with deletion from the repertoire of T cell clones expressing TCRs either unresponsive or overtly reactive to self-peptide major histocompatibility complexes (MHCs) (*9*, *10*). The interaction between the germline encoded HLA type and an individual’s TCR repertoire thus has substantial implications for our understanding of the pathophysiology of immune-mediated diseases and, additionally, where T cell immunity plays a key role in clinical responses to therapeutics. However, in the context of ICB therapy, the relationship between germline genetics, including classical HLA alleles, and TCR usage as well as any association to clinical response to treatment are largely unexplored.

Here, we have investigated the association between genetic factors and CD8^+^ TCR repertoire composition across 250 patients receiving ICB for cancer, integrating germline genotyping and TCR sequencing both before and post–ICB treatment. We describe a complex relationship between genotype determined class I HLA allele carriage and the TCR repertoire, specifically identifying a number of HLA-matched TCR chains. By exploring single-cell RNA sequencing (scRNA-seq) data from patients receiving ICB, we find that CD8^+^ T cell clones incorporating HLA-matched TCR demonstrate increased expression of tumor reactivity associated genes. Last, we find that the presence of HLA-matched TCR clones is associated with overall survival (OS) in patients receiving ICB for MM.

## RESULTS

### Chain usage and clonal stability of CD8^+^ TCR repertoire in ICB treatment

**Fig. 1.**
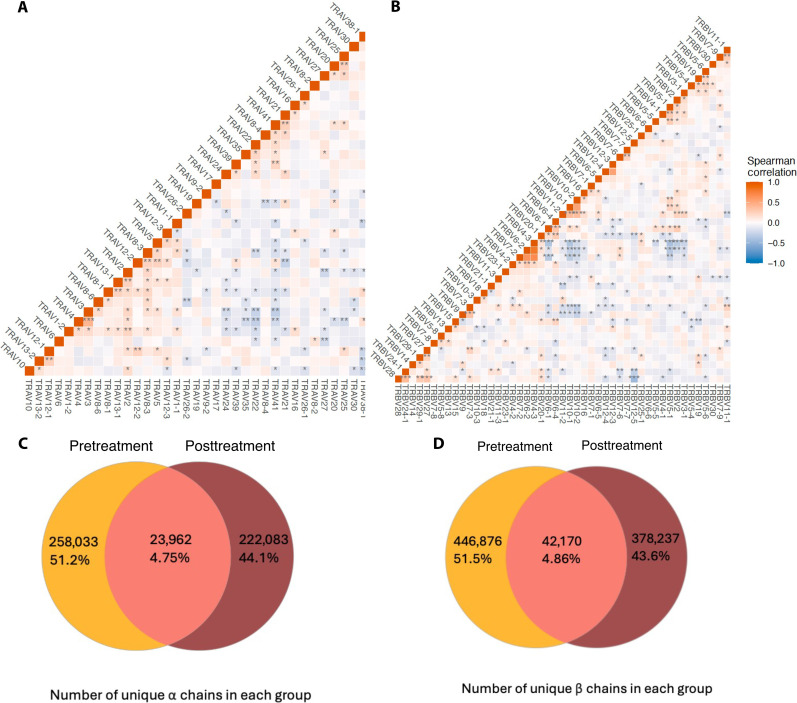
Summary of TCR chain relationships. Spearman correlation between each (**A**) TCRα and (**B**) TCRβ V-gene usage in our cohort. In each plot, V-gene usage is calculated by normalizing the number of unique clones per individual. *P < 0*.*05; **P < 0*.*01. (**C**) Number of TCRα chains and (**D**) TCRβ chains in three groups of cells: those seen only in individuals pre–ICB treatment, those seen in individuals only post–ICB treatment, and those seen both pre– and post–ICB treatment.

We performed RNA sequencing (RNA-seq) of peripheral CD8^+^ T cells isolated from PBMCs before the first and second cycles of ICB treatment from 250 patients receiving ICB for cancer (table S1). By performing intra-chain α and β chain V-gene usage correlation, we identified conserved patterns of V-gene repertoire frequency (Fig. 1, A and B). In addition, we performed inter-chain correlation analysis of α and β chain usage across the cohort, finding a generally limited relationship between chain usage by sample (fig. S1). The exception to this is the highly co-regulated usage of *TRAV1-2* and *TRBV6-4*, which was markedly distinct from other chains. *TRAV1-2* forms the key α chain TCR for MR1-ligated mucosal-associated invariant T (MAIT) cells (*11*), with *TRBV6-4* being the major β chain partner. Similarly, other β V-genes known to form MAIT cell TCR with *TRAV1-2*, namely, *TRBV6-1* and *TRBV20.1*, displayed higher correlations with *TRAV1-2* (*12*). Thus, it would appear that, while there is limited structure to the relationship between α and β V-gene paired CD8^+^ TCR chain usage, reactivity to MR1 versus HLA forms the leading determinant of the patterns observed.

We had paired pre– and post–ICB treatment RNA-seq data from 179 patients from which the TCR was mapped. This identified 504,078 unique α chain clones and 867,283 unique β chain clones. Analysis of chain conservation across ICB treatment demonstrated that only a small number of clones were resampled across both time points, likely reflecting the relatively small proportion of total TCR being sampled as well as ICB-induced T cell mitosis and changes in clonal abundance (Fig. 1, C and D) (*13*, *14*).

**Fig. 2.**
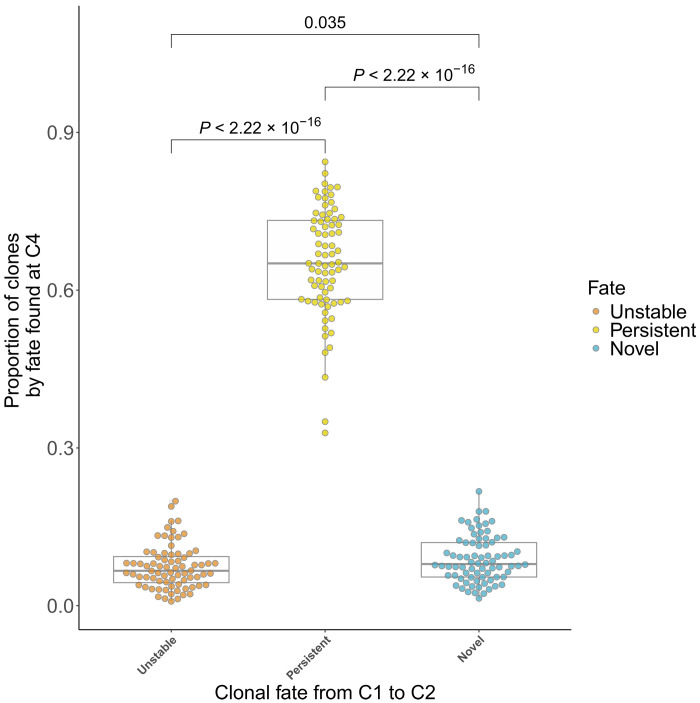
Assessment of clonal resampling at a later time point. For 76 of the 179 samples taken at C1 and C2, we also had a sample taken at the fourth cycle of ICB, C4. We explored the proportion of clones per individual that were sampled at C1, but not C2, being sampled at this later time point, unstable; the proportion of those novel at C2 resampled at C4; and the proportion of those persistent across C1 and C2 resampled at this later time point. *P* values derived from Wilcoxon rank sum test.

We have previously shown that a subset of CD8^+^ T cell clones demonstrates marked longevity in patients (*14*, *15*), suggesting that persistent clones were in some way different from those that changed with treatment. However, given the high degree of clonal flux post-ICB, as well as the inherent limitations on the number of clones that could be sampled, we wished to confirm that resampled clones displayed a different pattern of behavior in this large dataset. To do this, we analyzed samples from 76 of the 179 samples that, in addition to paired pretreatment (C1) and posttreatment (C2; taken immediately before the second cycle of treatment) data, we had a further CD8^+^ T cell sample taken before the fourth cycle of ICB (C4; days 63 to 84), where we also had TCR mapped from bulk RNA-seq data. For each sample, we explored the proportion of clones that were not resampled from C1 to C2, referred to as “unstable,” resampled at this later time point, as well as the proportion resampled of those observed posttreatment only, “novel,” and the proportion resampled of those that were present across both samples, defined as “persistent.” Across patients a median proportion of 0.0664 [interquartile range (IQR), 0.0439 to 0.0935] of unstable clones were resampled at C4, with a slightly higher number of novel clones redetected at 0.0793 (IQR, 0.0545 to 0.120, *P* = 0.035 versus unstable, Wilcoxon rank sum test). Conversely, we noted that a median proportion of 0.651 (IQR, 0.583 to 0.733) of persistent clones was again detectable at this much later time point (*P* < 2.2 × 10^−16^ versus unstable and *P* < 2.2 × 10^−16^ versus novel; Fig. 2), demonstrating that these clones were not randomly resampled but had highly divergent properties, including increased clonal persistence.

### TCR V-gene usage demonstrates both cis and trans genetic associations

**Fig. 3.**
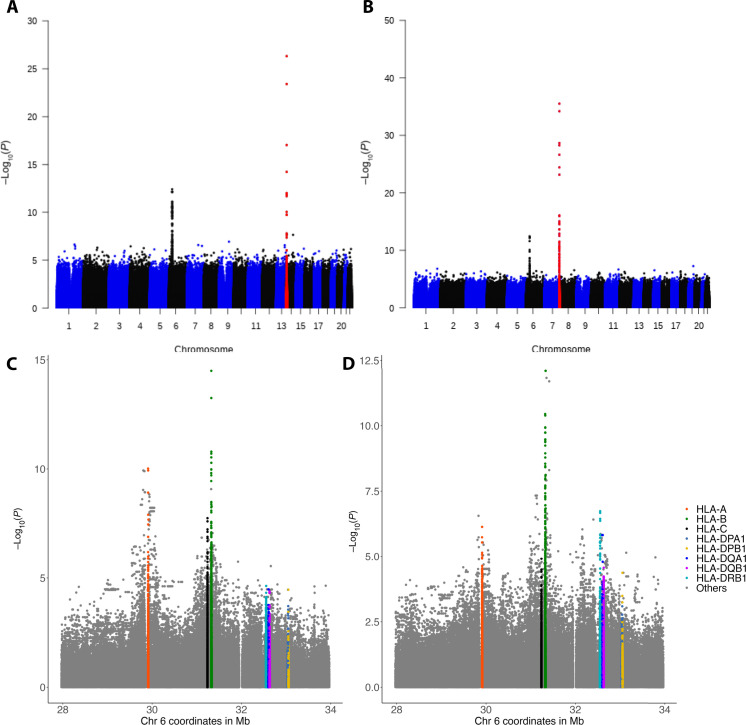
Association between TCR V-gene usage and germline genetic variation. (**A**) Manhattan plot of association between TCRα chain V-gene usage. The TRAV gene locus is highlighted in red. (**B**) Manhattan plot of TCRβ chain V-gene usage. The TRBV gene locus is highlighted in red. The strength of association is indicated by the −log_10_ of the *P* value of the linear model fitted between each V-gene usage and each variant on the *y* axis. The *x* axis shows the genomic position (in GRCh38). All V-genes are plotted together, and TRAV and TRAV gene loci are highlighted in red. Locus plot of association between (**C**) TRAV and (**D**) TRBV usage and variants in the MHC region. Classical HLA genes are annotated with different colors in the legend.

We proceeded to explore the relationship between genetics and TCR usage. No difference in overall V-gene usage pre- and posttreatment was observed in principal components (PCs) analysis (fig. S2), and, henceforth, we focused our association analysis on pretreatment samples where our power was greatest to detect effect (*n* = 250). Genome-wide genotyping was performed across samples, resulting in 486,469 single-nucleotide polymorphisms (SNPs) available for post–quality control (QC) analysis. We defined V-gene usage for α and β chains as normalized count of unique clones per V-gene and modeled this as a quantitative trait. For each V-gene, we performed additive linear regression to identify genetic variants associated with V-gene usage, correcting for the first two genetic PCs (to control for population structure), two TCR PCs (corresponding to confounding variation), age, sex, and cancer type (figs. S3 and S4). To account for multiple testing and also correlation between V-gene usage, we permuted the phenotype dataset 1000 times, preserving individual level V-genes but reordering the samples, resulting in a permutation *P* value threshold of 3.83 × 10^−9^ for β chain V-gene (TRBV) and 3.46 × 10^−9^ for α chain V-gene (TRAV; fig. S5, A and B). In total, we performed genome-wide association studies for 47 β chain and 42 α chain V-genes from pre–ICB treatment samples. After running >40 million regression models, we observed, as per previous observations (*16*), strong cis associations to a subset of the α and β chains, corresponding to their respective genomic loci (Fig. 3, A and B). Additionally, we noted a strong secondary trans-acting signal to both chains corresponding to the MHC region at 6p23.

The mechanisms whereby cis-acting polymorphisms may influence V-gene usage may be similar to standard expression quantitative loci, with polymorphisms in regulatory regions such as promoters and enhancers regulating expression (*17*, *18*). However, by providing equal weighting to clones irrespective of numbers of copies detected, our approach gives insights into propensity for clonal formation. Thus, cis-acting variants are more likely to influence propensity to recombination, defined by the local three-dimensional structure of the loci (*19*, *20*). In total, 11 different TRBV genes were associated with variants in cis at *P* values below the permuted significance threshold (table S2). The most significant association was for *TRBV28* with rs4726571, 3.7 kb upstream (β = −0.957, *P* = 2.12 × 10^−40^). At the α locus, only *TRAV26-2* and *TRAV38-1* demonstrated associations with clonal usage after correcting for multiple testing, the most significant being with rs1023437, which is 41 kb downstream from *TRAV26-2* (β = −1.035, *P* = 4.88 × 10^−27^). We performed further conditional analysis on all the significant V-genes, controlling for primary cis signals. Only one secondary signal was observed to *TRBV4-3*, with the lead SNP being rs361489 (β = 0*.*571, *P* = 9.83 × 10^−12^), and peak secondary signal to rs17249 (β = −0*.*460, *P* = 2.66 × 10^−11^), indicating multiple germline variants determining the usage of this V-gene.

### V-gene usage is associated with classical HLA alleles

We observed a trans locus associated with both TRAV and TRBV genes that maps to 6p23, which corresponds to the MHC region. To further define the associations, we tested V-gene usage with imputed SNPs in the MHC region as well as classical HLA alleles and coding polymorphisms. Classical class I HLA alleles (HLA-A, HLA-B, and HLA-C) and class II alleles (HLA DRB, DQA, DQB, DPA, and DPB) were imputed to four-digit resolution. In total, we tested 16,781 SNPs, 174 classical alleles, and 2119 amino acids. To control for false positives, we performed a permutation analysis by randomly shuffling the phenotypes and fitting the regression model 1000 times deriving empirical *P* value thresholds of 3.37 × 10^−7^ for β chain and 3.61 × 10^−7^ for α chain V-genes, respectively (fig. S5, C and D). In total, we observed five β chain V-genes and five α chain V-genes that had significant associations with variants within the MHC region (Fig. 2, C and D; table S3; figs. S6 to S8).

**Fig. 4.**
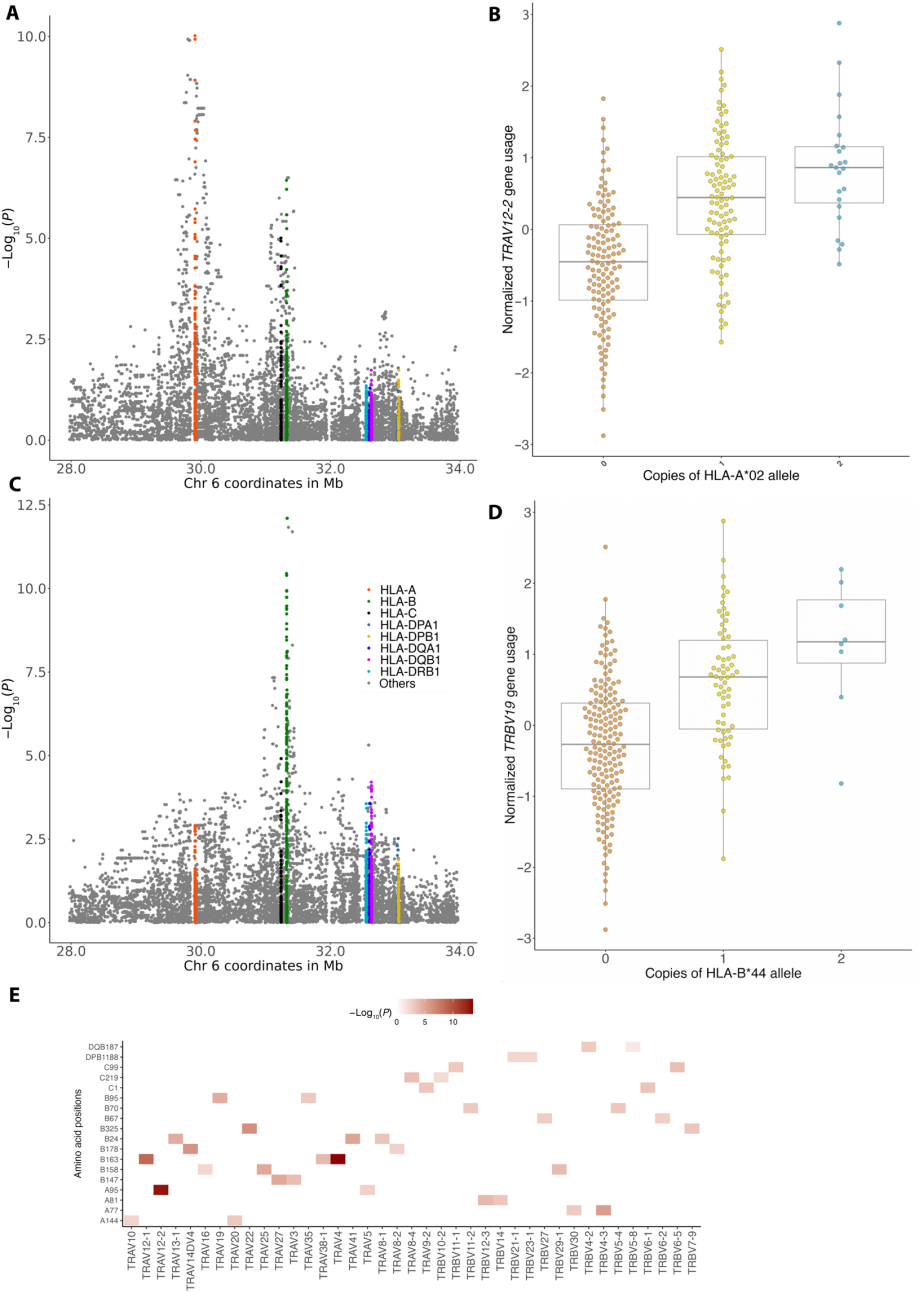
Association between V-gene usage and MHC variants. The MHC locus plot of (**A**) *TRAV12-2* and (**C**) *TRBV19*. The *x* axis shows the genomic positions of chromosome 6 (build GrCh38), and the *y* axis is the −log_10_(*P*) obtained from two-sided regression analyses. (**B**) *TRAV12-2* usage plotted against dosage of the most significant classical HLA allele association, HLA-A*02. Each dot represents an individual. (**D**) *TRBV19* usage plotted against dosage of the most significant classical HLA allele, HLA-B*44. Box plots show median (horizontal bar), 25th and 75th percentiles (lower and upper bounds of the box, respectively) and 1.5× IQR (or minimum and maximum values if they fall within that range; end of whiskers). (**E**) Heatmap of omnibus test of each HLA amino acid position against V-gene usage.

We found that the most significant association in the α chain was HLA-A*02 with *TRAV12-2* (β = 0.689, *P* = 3.04 × 10^−13^; Fig. 4, A and B). A strong bias toward *TRAV12-2* usage has been described for melanocyte antigen (Melan-A)–specific TCR, with predominant interaction between the *TRAV12-2* chain and the HLA-A2/Melan-A peptide located in the CDR1 loop (Gln31) (*21*). Given that Melan-A is one of the most common melanoma associated antigens, these results suggest that germline determined *TRAV12-2* usage may play a role in anti-melanoma immunity. There were no secondary signals in the conditional analysis of any of the TRAV genes. The strongest association observed to the β chain was between *TRBV19* and rs2250287 (β = 0.760, *P* = 7.93 × 10^−13^; Fig. 4C) with the top classical HLA allele associated with *TRBV19* being HLA-B*44 (β = 0.734, *P* = 1.83 × 10^−10^; Fig. 4D) in linkage disequilibrium with rs2250287 [coefficient of determination (*R*^2^) = 0.882]. At four-digit HLA resolution, *TRBV19* was associated with both HLA-B*44:02 (β = 0.576, *P* = 2.78 × 10^−5^) and HLA-B*44:03 (β = 0.725, *P* = 1.23 × 10^−4^). HLA-B*44:02 is associated with protection against multiple sclerosis in several studies (*22*–*24*), while, in the context of melanoma, carriage of the B*44 supertype has been associated with improved outcomes to ICB treatment (*25*), although this was not reproduced in a follow-up study (*26*). Notably though, as per *TRAV12-2*, *TRBV19* is enriched for TCR in T cells reactive to Melan-A in patients with MM (*21*). We further performed conditional analysis and observed only one secondary signal among the significantly associated V-genes, *TRBV19*, with the top secondary SNP being rs62395263 (β = 0.705, *P* = 2.96 × 10^−7^). Last, we explored whether we could see consistent direction of effect in a further independent cohort of genotyped patients receiving ICB for cancer obtained post–primary analysis where we had pretreatment CD8 TCR mapped from RNA-seq data (*n* = 34). This provided strong supportive data of the primary observations, with 10 of 11 showing consistent direction of effect and 8 of 11 showing significant association (*P* < 0.01, Wilcoxon-test; fig. S9). Additionally, despite the replication dataset being very underpowered for trans effects, three of the five tested trans observations from the primary analysis showed consistent directional effects, and the other two did not have enough minor allele carriers to test (figs. S10 and S11).

To determine whether specific HLA amino acid positions were associated with V-gene usage, we tested variable amino acid positions by grouping haplotypes carrying a specific residue at each position in an additive model (table S4 and fig. S12). We found that the most significant association in α chain was between *TRAV12-2* and position 74 (exon2) in HLA-A (*P*_omnibus_ = 2.93 × 10^−13^), while the most significant association in β chain was between *TRBV19* and position 45 (exon2) in HLA-B (*P*_omnibus_ = 5.08 × 10^−11^). These positions had stronger association signals than any single SNP or classical HLA allele and fall within the peptide-binding groove of the respective HLA protein, indicating that variation in the amino acid content of the peptide-binding groove is the major genetic determinant of V-gene usage, likely reflecting presentation of a common antigen being the key driver (Fig. 4E).

### CDR3 amino acid *K*-nucleotide oligomers are associated with HLA alleles

In both α and β TCR chains, CDR3 loops, which form the hypervariable peptide contacting region, have the greatest diversity in sequence and are the crucial determinants of antigen binding specificity (*6*). We hypothesized that specific CDR3 sequences would demonstrate reciprocal association with variants in the MHC region. To test this, we used a *K*-nucleotide oligomer–based approach across TCR mapped from the CD8^+^ T cell bulk sequencing data, using a sliding window of seven amino acids to quantify *K*-nucleotide oligomer motif usage in the CDR3 region (6654 β chain and 2306 α chain *K*-nucleotide oligomers, respectively) across individuals. Linear models were subsequently used to test the association between CDR3 *K*-nucleotide oligomers and HLA alleles, incorporating the same covariates as prior analyses.

While no significant associations were identified for the α chain, we identified two significantly associated *K*-nucleotide oligomers in the β chain (fig. S13). The most significant β chain CDR3 *K*-nucleotide oligomer association was between TGDSNQP and HLA-B*35:01 (β = 1.41, *P* = 1.57 × 10^−13^), while the *K*-nucleotide oligomer TSGDYNE was associated with rs3763288 (β = 1.12, *P* = 2.66 × 10^−10^), an intronic variant in *MICA*, encoding MICA (MHC class I–like molecule A), a ligand for the natural killer (NK) and CD8^+^ T cell–activating immunoreceptor NKG2D (*27*), which triggers a cytotoxic response upon ligation (*28*). Cell surface release of MICA in a soluble form (sMICA) has been implicated in cancer cell escape from NK cell immune surveillance (*29*), and rs3763288 forms a quantitative trait locus for blood sMICA levels (*30*). To test whether the observed associations with CDR3 *K*-nucleotide oligomers were influenced by V-gene usage, we performed a correlation analysis between CDR3 *K*-nucleotide oligomers and V-gene usage. While no V-genes were associated with TGDSNQP, indicating that this *K*-nucleotide oligomer association is independent of V-gene pairing, TSGDYNE was associated with *TRBV19* [Pearson correlation coefficient (*r*) = 0.276, *P* = 9.61 × 10^−6^]. We repeated our analysis of *TSGDYNE* against rs3763288 conditioning on *TRBV19*, and the association remained significant (β = 1.01, *P* = 1.06 × 10^−8^), suggesting that, while TSGDYNE was frequently observed in CDR3 from *TRBV19* containing TCR, the association between TSGDYNE and rs3763288 is not dependent upon *TRBV19*.

### Genetically associated TCR display shows CD8^+^ T cell subset preference

To provide further granularity to these associations, we performed scRNA-seq with V(D)J mapping on CD8^+^ T cells from 59 individuals with melanoma, 55 of whom were also included in the bulk RNA analysis (total, 48,246 T cells; median, 330 cells per patient; IQR, 158 to 1006 cells). A further strength of this approach is that it enabled examination of cell-type–specific associations across T cell subsets (table S5).

Testing the lead β chain cis association, rs4726571 with *TRBV28*, demonstrated replication at the single-cell level across all CD8^+^ cells (β = −0.339, *P* = 0.00412) and was also observed in all CD4^+^ cells (β = −0.320, *P* = 0.00551), indicating a pan T cell mechanism. Notably, the association was stronger in CD8 terminally differentiated effector memory cells reexpressing CD45RA (TEMRA) cells (β = −0.489, *P* = 5.53 × 10^−5^) and CD8^+^ T effector memory (TEM) cells (β = −0.426, *P* = 1.35 × 10^−4^) compared to that in CD8 naïve cells (β = −0.236, *P* = 0.0700), suggesting additional maintenance of clonal abundance due to antigen recognition.

In general, associations in trans are harder to replicate due to the typically secondary mechanisms of action. Given this, it was noteworthy that we observed the same direction of effect of the leading MHC × V-gene association (table S6) between *TRBV19* and HLA-B*44 across all CD8^+^ T cells (β = 0.0823, *P* = 0.391). In keeping with this being secondary to antigen presentation via class I HLA to CD8^+^ cells, we found no evidence of association to CD4^+^ T cells (β < 0). Further, in keeping with this association being secondary to antigen presentation, the association across CD8^+^ T cell subsets demonstrated a stronger signal in CD8 TEMRA cells (β = 0.373, *P* = 0.0398) compared to that in CD8 naïve cells (β = 0.0119, *P* = 0.950).

### ICB alters HLA:V-gene associations

**Fig. 5.**
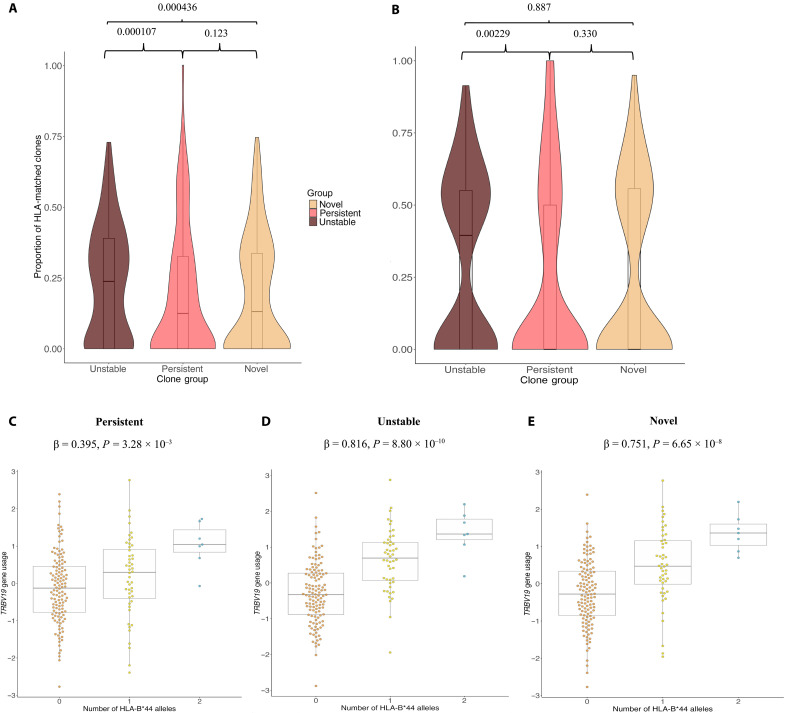
HLA:V-gene association across ICB treatment. The proportion of HLA-matched clones in three groups of clones: those only observed pretreatment (unstable), those observed posttreatment only (novel), and those present across both samples (persistent) in (**A**) β chain and (**B**) α chain. Each dot represents an individual and the paired Wilcoxon test *P* values are represented above the plots for comparison between the groups. *TRBV19* plotted against HLA-B*44 in (**C**) persistent, (**D**) unstable, and (**E**) novel clones, demonstrating differential relationship between V-gene usage and copies of classical HLA alleles.

Our findings regarding the frequency of V-gene usage and HLA allele status were based on CD8^+^ T cells from patients before ICB treatment. We wanted to explore the effect of ICB treatment on potential HLA–V-gene selection. A key strength of the dataset is that most patients had paired CD8^+^ T cell samples before and after their first cycle of ICB treatment, allowing us to explore genotype × V-gene usage posttreatment. Focusing on the 179 individuals for which paired samples were available, we subdivided clones into the previously defined unstable, novel, and persistent. We selected all V-genes that had a significant association with variants in the MHC region (table S3) and extracted the top classical HLA alleles with that V-gene. We defined a clone to be HLA matched if it had both the presence of the associated HLA allele and V-gene. We subsequently calculated the proportion of HLA-matched clones for each individual within each clone group. For the β chain, we found that the proportion of HLA-matched clones in each individual in the unstable group was significantly higher (median, 0.238) than in the novel group (median, 0.132; *P* = 0.000436; Wilcoxon test) and the persistent group (median, 0.125; *P* = 0.000107; Wilcoxon test; Fig. 5A). Although the median proportion of HLA-matched clones was higher in the novel group compared to that in the persistent group, this was not statistically significant. Similarly for the α chain, the proportion of HLA-matched clones was also higher in the unstable group than in the persistent group (*P* = 0.00229), although there was no significant difference in proportions between either unstable or persistent and novel groups (Fig. 5B). The observation that the median proportion of HLA-matched clones is highest in the unstable group suggests that, in the context of ICB therapy that acts on exhausted T cells, clones that grow and shrink with treatment, thus potentially responding to antigen presentation, are more likely to be HLA-selected (table S7). This is illustrated in the leading β chain association between *TRBV19* with HLA-B*44, which was strongest in the unstable and novel groups (Fig. 5, C to E).

### Cells with HLA-matched V-genes demonstrate increased tumor reactivity expression profiles

**Fig. 6.**
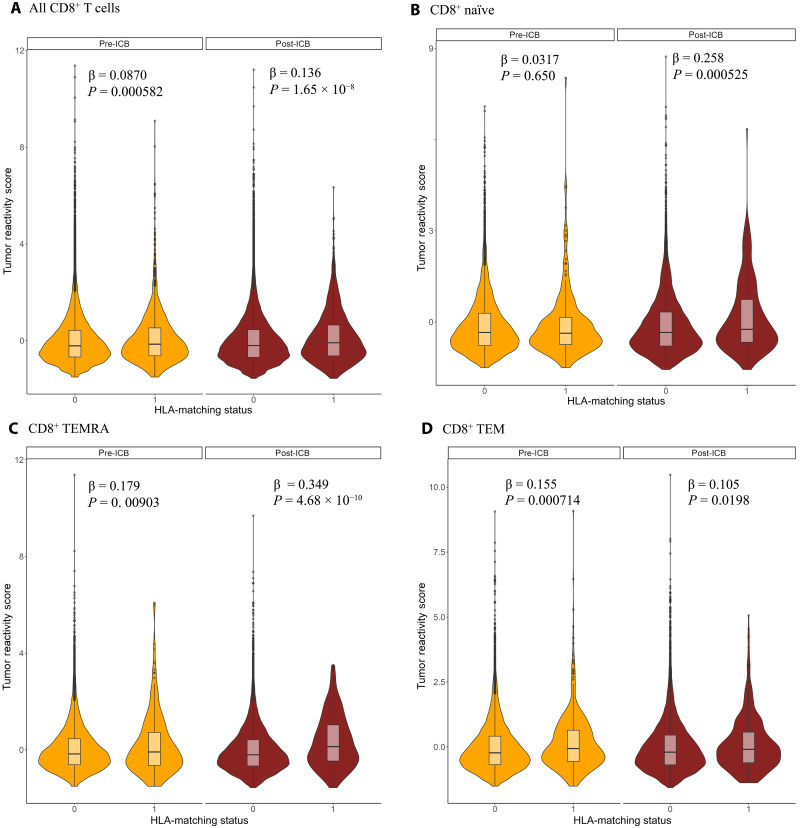
Association between V-gene usage and HLA-matching status. (**A**) HLA-matching status (0, unmatched; 1, matched) plotted against tumor reactivity score (TRS) for all CD8^+^ cells pre- and posttreatment with each dot representing one cell. The subplots are annotated with analysis of variance (ANOVA) *P* values comparing the model with HLA-matching status with the null model without HLA-matching status. (**B** to **D**) In each subplot, TRS is plotted against HLA-matching status with each dot representing one cell. Each subplot represents a CD8^+^ cell subset, showing that the largest difference occurs within T effector memory (TEM) cells before treatment but within terminally differentiated effector memory cells reexpressing CD45RA (TEMRA) cells after treatment.

Given that unstable clones are more likely to carry TCRs that demonstrate HLA selection, we sought to determine whether such HLA-selected clones showed evidence of tumor reactivity. To do this, we applied a set of 20 genes identified to characterize tumor-reactive cells based on single-cell sequencing in 59 patients with melanoma (*31*), generating a tumor reactivity score (TRS; table S8). Using V-genes significantly associated with variants in the MHC region, we extracted the top classical HLA allele associated with each V-gene and dichotomized cells according to whether they carried HLA-matched TCRs or not. For each cell, we calculated its TRS and determined the association between TRS and HLA-matching status using a linear model. We showed that HLA-matched pre-ICB CD8^+^ cells displayed a significantly increased TRS (β = 0.0870, *P* = 5.82 × 10^−4^), in keeping with cancer-related antigenic pressure in these patients. We subsequently explored whether the increased TRSs seen in HLA-matched cells were also seen in predefined cell subtypes (Fig. 6). Pretreatment, the most robust association was observed in T effector memory cells (CD8 TEM, β = 0.155, *P* = 7.14 × 10^−4^; table S9). To see whether this effect was driven by singlet or expanded clones, we tested this association across both categories, finding that the association between TRS and HLA-matching status was confined to cells from expanded clones (singlets: β = 0.0415, *P* = 0.256; expanded clones: β = 0.140, *P* = 6.87 × 10^−5^). Consistent with this observation, we found that TRS was positively correlated with clone size (*P* = 7.21 × 10^−34^, Spearman *r* = 0.0558).

To investigate whether the observed associations were different in posttreatment samples, we repeated this analysis for all cells from a subset of 56 individuals after the first cycle of ICB treatment. We demonstrated that the association between TRS and HLA matching among all CD8^+^ cells was far stronger post-ICB (β = 0.136, *P* = 1.65 × 10^−8^) and was also detectable across multiple cell subsets, with the strongest association in CD8 TEMRA (β = 0.349, *P* = 4.68 × 10^−10^), followed by naïve cells (β = 0.258, *P* = 5.25 × 10^−4^) and CD8 TEM cells (β = 0.105, *P* = 0.0198). We also separated the cells into whether they were singlets or from expanded clones, observing that, post-ICB, both singlets and expanded clones demonstrated increased TRS if HLA matched (singlets: β = 0.150, *P* = 3.85 × 10^−5^; expanded: β = 0.130, *P* = 7.18 × 10^−5^).

We noted that the strongest association between tumor reactivity and the HLA associated V-genes was observed in CD8 TEM cells before ICB treatment and in CD8 TEMRA cells after treatment. This shift may be linked to previously described phenomenon of ICB-induced clonal shuffling between these subsets (*2*). Additionally, the increase in TRS in HLA-matched naïve cells post-ICB aligns with the findings in singlet clones, suggesting the expansion of de novo clones driven by tumor antigen and ICB treatment.

### Carriage of HLA-matched clones is associated with improved OS

Given that, in the single-cell data, HLA-matched clones were observed to demonstrate a higher TRS, we proceeded to examine the relationship between carriage of HLA-matched clones and long-term oncological outcomes. Survival analysis across all patients receiving ICB for metastatic disease (*n* = 231) for which we had pretreatment samples demonstrated that patients demonstrating carriage of HLA-matched clones before treatment had improved OS (*P* = 0.0399, log-rank test; fig. S14A).

**Fig. 7.**
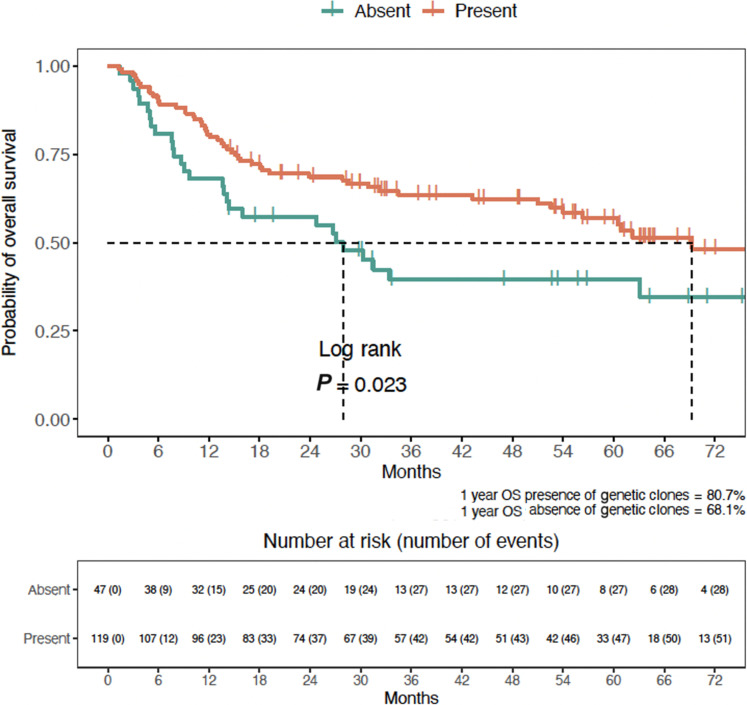
Relationship between OS and presence of HLA-matched clones. Kaplan-Meier plot denoting that OS is longer in patients who have a HLA-matched clone either before or after first cycle of ICB treatment.

Similarly, we examined samples for which we had both pre- and posttreatment data, finding that 130 of the 179 patients had HLA-matched clones on at least one of these two time points. While the proportion of HLA-matched clones frequently changed across patients with ICB, no patients were found to develop HLA-matched clones posttreatment who did not already have pretreatment matched clones, with 127 of the 130 (97.6%) patients displaying one or more HLA-matched clones across both time points, in keeping with the importance of the pretreatment state for ICB activity. Notably, analysis of these patients again showed that carriage of HLA-matched clones at any time point was associated with improved OS (*P* = 0.0229; Fig. 7), with a similar effect observed when analysis was confined to those with MM (*P* = 0.0553; fig. S14B).

## DISCUSSION

Here, we present the first analysis of genetic determinants of CD8^+^ T cells in patients with cancer who are undergoing treatment with ICB. We describe associations both in cis to the V-genes of α and β TCR chains and trans-acting associations that map to the MHC and can reduce to individual class I HLA alleles. In addition to describing the relationship between germline variation and the CD8^+^ TCR repertoire in patients with cancer, our findings support an interaction between genetic effects on the repertoire and ICB treatment with evidence that this relationship has prognostic implications.

Given that our phenotype consists of unique flattened clones per individual, providing equal weighting to clones that exist as singlets and those expanded, polymorphisms associated in cis to the *TRAV* and *TRBV* genes likely reflect influences on V(D)J recombination propensity of clone that are selected for in that individual. Conversely, trans associations between MHC polymorphisms and HLA alleles with V-gene usage are proposed to reflect indirect pressures upon clonal deletion or maintenance through clonal selection via antigen presentation. In health, the drivers of this selection are both central thymic deletion of autoantigen reacting TCRs (*32*) and peripheral selection of antigen-reactive TCR (*33*). In the context of cancer, where the burden of chronic antigen presentation leads to immune exhaustion and also during ICB treatment, which releases immune checkpoints and favors autoimmunity causing immune-related adverse events, our data suggest that the major determinant of selection is successful antigen presentation to specific TCR clones. This is supported by the increased strength of association that we observed for de novo versus persistent clones post-ICB and also the relative strength of associations in CD8^+^ TEMRA cells versus CD8^+^ naïve cells. Why non–HLA-selected clones are more likely to be persistent across ICB treatment is unclear, one possibility being they are reactive to chronic viral infection and non-exhausted. This likely speaks as to the complex processes in TCR recognition of antigen versus the availability of specific antigens.

Our dataset is unique in that we have pre- and posttreatment samples from the same individuals. As such, we are able to evaluate the effect of ICB on cells expressing V-genes that show association with selection to the HLA type of the individual they come from. However, the relatively small sample set means that only the most significant HLA:TCR V-gene relationships are discernible in these data. Nonetheless, analysis of scRNA-seq data from the same individuals demonstrates that cells carrying TCRs that show association to the HLA of the individual from which they are obtained display gene expression profiles, indicative of increased tumor reactivity compared to HLA-unmatched cells. Notably, this difference is more significant post–ICB treatment, suggesting ICB enhancement of tumor reactivity in these cells. Last, we are able to show that patients with HLA-matched clones demonstrate increased OS, reflecting a direct HLA:TCR selection impact on cancer survival post-ICB.

While the paired collection of samples from patients with cancer pre- and post-ICB means that the dataset provides unique insights into the relationship between genotype, ICB treatment, and TCR repertoire, we have only characterized the CD8^+^ subset. It is recognized that CD4^+^ T cells also play a role in the ICB-induced anticancer response, especially with respect to the development of autoimmune toxicities (immune-related adverse events). Given the relative enrichment of class II HLA genes with autoimmune phenotypes, future studies exploring the relationship between TCR repertoire and genotype of CD4^+^ T cells in patients with cancer will be of high importance.

In conclusion, we present a study of the germline genetic association of CD8^+^ TCR repertoire within patients receiving ICB for cancer, showing significant associations at the MHC region with genes that are cancer relevant. Notably, these associations are influenced by ICB treatment and demonstrate a relationship with patient survival outcomes. 

## METHODS

### Participants

Patients were recruited from Oxford University Hospital when they were referred to receive ICB as therapy for melanoma, renal cell carcinoma, and colorectal cancer. All patients provided written informed consent to donate samples to the Oxford Radcliffe Biobank (Oxford Centre for Histopathology Research ethical approval reference 19/SC/0173, project nos. 16/A019, 18/A064, and 19/A114) and allow access to their clinical data. Patients received either combined ICB (ipilimumab 3 mg/kg plus nivolumab 1 mg/kg every 3 weeks for ≤4 treatment cycles, followed by maintenance 480 mg nivolumab every 4 weeks) or single ICB consisting of either 480 mg nivolumab every 4 weeks, 200 mg pembrolizumab every 3 weeks, or 400 mg pembrolizumab every 6 weeks. Patient demographic and clinical characteristics were collected from the electronic health record system.

### Sample collection

Blood (30 to 50 ml) was collected into EDTA tubes (BD Vacutainer system) just before administration of the first cycle of ICB. The blood was centrifuged to obtain peripheral blood mononuclear cells (PBMCs) and plasma by density centrifugation (Ficoll-Paque). CD8^+^ T cells were isolated from PBMCs by CD8-positive selection using anti-CD8 antibody–conjugated magnetic beads (Miltenyi, catalog no. 130-045-201).

### RNA extraction

Cells were resuspended in 350 μl of RLT Plus buffer supplemented with β-mercaptoethanol or dithiothreitol. QIAshredder (QIAGEN) was used to homogenize the sample, and an AllPrep DNA/RNA/miRNA kit (QIAGEN) was then used for DNA/RNA extraction. RNA was then eluted into 34 μl of ribonuclease-free water with concentration quantified by Qubit, and DNA was eluted into 54 μl of elution buffer. Both RNA and DNA samples were subsequently stored at −80°C until sequencing and genotyping.

### Bulk RNA-seq

RNA was thawed on ice before mRNA isolation using Poly(A) mRNA Magnetic Isolation Module kits (NEBNext). Up to 600 ng of RNA was then used to generate double-stranded DNA libraries using NEBNext Ultra II Directional RNA Library Prep Kits as previously described (*14*). Samples were then sequenced on either an Illumina HiSeq4000 [75–base pair (bp) paired-end] or a NovaSeq6000 (150-bp paired-end). We performed TCR analysis using the MiXCR package (*34*) with settings as previously described (*2*, *14*).

### Single-cell RNA and TCR sequencing

scRNA-seq data were acquired from two different experiments. The first used fresh PBMCs and has been previously published (*2*). The second, obtained from cryopreserved PBMCs, was processed using two separate protocols. In the first protocol, dead cells were first incubated with live:dead magnetic beads (Miltenyi dead cell removal kit) and run over a MACS LD column to remove dead cells. A total of 60,000 live PBMCs were then loaded into the partitioning reaction and processed using 5′ single-cell RNA and V(D)J sequencing kits (version 2, 10X Genomics, Pleasanton, CA) following the manufacturer’s protocols. In the second protocol, CD8^+^ T cells were first isolated by incubating with live:dead and CD8^+^ T cell–negative selection beads and run over a magnetic column. A total of 60,000 CD8^+^ T cells were taken from the flow-through and processed as above.

Sequencing of single-cell libraries were performed on an Illumina NovaSeq 6000 S4 flow cell (150-bp paired end). For each pool, there were five library samples: PBMC gene expression (GEX) libraries, CD8^+^ T cell GEX libraries, PBMC origin TCR libraries, PBMC origin B cell repertoire libraries, and CD8^+^ T cell origin TCR libraries. Batch 1 of sequencing was the first four pools that were sequenced across one lane of an S4 flow cell. This was deliberately under-sequenced to establish sequencing requirements across the rest of the samples. Following this sequencing run, there were a median of 18,000 reads per cell for GEX libraries (target, 27,500) and 4800 reads per cell for V(D)J libraries (target, 7000). We therefore sequenced the remaining seven pools plus additional sequencing of libraries from the first four pools that were under-sequenced, across three further lanes of an S4 flow cell. Across both runs, we achieved a median of 28,500 reads per cell for the GEX libraries and 7100 reads per cell for the TCR libraries.

Single-cell sequencing data were aligned against the GRCh38 human reference genome using Cellranger (v6.0.1) for GEX libraries and Cellranger VDJ for V(D)J libraries. For samples that had been sequenced across multiple lanes, the FASTQ files were inputted for alignment on the same Cellranger run. EmptyDrops (*35*) was used to identify empty droplets (false discovery rate, 0.01). Barcodes were excluded only if they were called as empty by both Cellranger and EmptyDrops. Cellsnp-lite (*36*) was used to reconstruct genotype data from reads, and, then, Vireo was used to compare this to patient-level genotype to de-multiplex samples from pools. Doublet identification was performed during sub-clustering by identification of mixed transcriptomes of canonical markers. Cells with <300 transcripts and >20% of mitochondrial-encoded genes were removed.

### Single-cell sequencing integration and annotation

scVI (*37*) was used for the integration of the two single-cell experiments. We first selected 4000 highly variable genes, from the whole PBMC dataset in Scanpy (*38*). An scVI model was then trained using the pool label as the batch variable and the following model parameters: number of latent variables of 30 and batch size of 1024. Once the core model was trained, datasets containing enriched cell types or higher mitochondrial proportions (10 to 20%) were further referenced mapped using scArches (*39*). Next, clustering was implemented on a nearest neighbor graph using the 30 latent dimensions that were obtained from the scVI and scArches output. Here, the number of neighbors was set to *k* = 30 and distance metric was set to “cosine.” We then performed coarse Leiden clustering on the graph with resolution *r* = 0.03. For each of the resulting level 1 clusters, we calculated a new neighbor graph using scVI’s 30 latent dimensions, with the number of neighbors again set to *k* = 30. On the basis of the new neighbor graph, each cluster was clustered into smaller “level 2” clusters with Leiden clustering at resolution *r* = 0.3. Level 2 clusters were then annotated on the basis of differentially expressed genes into broad cell-type categories: “CD8NK,” “CD4,” “B cells,” and “myeloid platelets.”

To fully capture cell-type heterogeneity, we remodeled each broad level 2 subset. Per level 2 cell type, we calculated 4000 highly variable genes, retrained scVI models using the same parameters, and calculated new neighbor graphs. To guide clustering, we first performed automated cell-type annotations on scVI embeddings with CellTypist (*40*). Cluster-specific marker genes were identified by performing differential expression analysis in Scanpy on a given cell type compared to the rest of the cells from the same level annotation. Where clusters were highly similar, we merged them on the basis of hierarchical dendrograms generated from gene expressions. For final CD8^+^ T cell annotations, cross-validation was performed on the basis of assigned annotations from the published dataset (*2*).

### QC and HLA imputation

Genotyping was performed on the Illumina Global Screening Array 24 v3 (Illumina) using build hg38. For sample QC, we used the following thresholds: heterozygosity outside 3.5 SDs and missing > 10% of SNPs. All samples were unrelated as assessed by inheritance by descent (IBD), with the max IBD between a pair of samples being 0.04. We did not note ancestry outliers (fig. S3). A total of 250 individuals were included including 231 patients with melanoma, 17 with renal cell cancer, and two with colorectal cancer (table S1). For SNP QC, we used the following thresholds: MAF of 0.01, Hardy Weinberg equilibrium of 0.000001, and present in at least 90% of individuals, resulting in a total of 486,469 SNPs tested for the genome-wide association study. For HLA imputation, we used the Michigan Imputation Server version 2 multi-ethnic panel to validate, resulting in a total of 2212 amino acids, 265 classical HLA alleles, and 17,952 SNPs imputed. This uses build hg37, but, for associated variants, we provide corresponding hg38 coordinates, the conversion between builds being performed using LiftOver (https://liftover.broadinstitute.org). The same QC thresholds were applied to the HLA imputed data.

### Association between TCR and genetic germline variation

Individual TCR sequences were mapped from bulk CD8^+^ T cell RNA-seq data using MixCR (*34*), and features were extracted, namely, V-gene usage, clone counts, and CDR3 amino acid sequences. Only productive clones were included. To obtain the V-gene usage phenotype, the number of unique clones with V-gene was counted, and, then, normalization was performed by trimmed mean of *M* values (TMM) function in edgeR (version 3.18) (*41*) followed by batch correction using removeBatchEffect function. After that, inverse normal rank transformation (INRT) was performed for each phenotype across the samples. A linear regression model was fitted in Plink (version 2.0) (*42*) to test each V-gene phenotype against genome-wide SNPs, HLA classical alleles, and amino acids/SNPs in the HLA region. We included two genetic PCs, two TCR PCs, age, sex, and cancer type as covariates

$$\begin{array}{c} V-\text{gene usage}∼\text{genetic variant}+\text{two}\;\text{genetic}\;\text{PCs}\\ +\text{two}\;\text{TCR}\;\text{PCs}+\text{age}+\text{sex}+\text{cancer type} \end{array}$$

### Permutation analysis

To determine an appropriate *P* value threshold for significance, we permuted it 1000 times scrambling the phenotype while keeping the same V-gene within each individual. The 5% percentile of *P* value was then chosen as the significance threshold. In addition to testing each HLA allele separately, we also conducted an omnibus test for each amino acid position where there is more than one amino acid present in the samples to investigate whether there are particular amino acid positions that are more associated to V-genes more strongly than classical HLA alleles.

### *K*-nucleotide oligomer analysis

To analyze the CDR3 amino acid sequences, *K*-nucleotide oligomers of length 7 were constructed using a sliding window approach across the sequence. We chose to use seven amino acids because the immunoreceptor tyrosine-based activation motif (ITAM) that is critical for the initiation of signaling following ligand engagement has a sequence of six to eight amino acids in its center (*43*).

This approach has been used successfully in other studies on TCRs (*44*, *45*). Only sequences between length 12 and 18 were included for this analysis. The *K*-nucleotide oligomers were also batch corrected and then TMM normalized followed by INRT normalized by the same procedure as the V-gene phenotype. The same linear model was fitted against HLA alleles as with the V-gene phenotype.

### Comparing pre- and posttreatment clones

Focusing on the 179 individuals for which paired samples (before and after first cycle of ICB) were available, we subdivided clones into three groups: those only observed pretreatment (unstable), those observed posttreatment only (novel), and those present across both samples (persistent).

Using V-genes significantly associated with variants in the MHC region, we extracted the top classical HLA allele associated with each V-gene. HLA-matched clones were defined as clones that had this classical HLA–V-gene pairing. HLA-unmatched cells were defined as all other clones. We then calculated the proportion of HLA-matched clones for each individual within each clone group (unstable, persistent, and novel).

To investigate whether ICB alters the proportion of HLA-matched clones, we performed a paired Wilcoxon test on proportions of HLA-matched clones between unstable and novel groups. For each of these three groups, we performed a V-gene-versus-HLA association analysis as described above for the significantly associated V-gene HLA pair.

### Single-cell TCR sequencing analysis

There were a total of 59 individuals for which we had these data as well as genotyping. Cells were included only if they had either one-α and one-β chain or two-α and one-β chain. Clones were collapsed to include only counts of unique clones and then normalized by total number of clones for the cell type being studied using the same method as bulk data. A variable representing experimental protocol was constructed. Because the experimental protocol was highly correlated with the TCR PCs, experimental protocol was regressed from the first two PCs and the residuals extracted. Then, a linear model was fitted between V-gene usage and HLA alleles with age, sex, cancer type, two genetic PCs, the residuals of two TCR PCs, and experimental protocol being the covariates. This was done individually for each cell type.

Single-cell gene expression sequencing was processed using Scanpy V1.10 (*38*) for the purpose of calculating TRS. Cells from each experimental protocol were normalized, log transformed, and then scaled. To calculate TRS for each cell, we took the sum of the 20 genes (*31*) and scaled it to have a mean of 0 and an SD of 1. We then divided the cells into two groups: those that were HLA selected and those that were not. Using V-genes significantly associated with variants in the MHC region, we extracted the top classical HLA allele associated with each gene. HLA-matched cells were defined as cells that had this classical HLA–V-gene pairing. Non–HLA-matched cells were defined as all other cells. To determine whether TRS was higher or lower in HLA-matched cells, we performed regression of TRS score against HLA-matching status with experimental protocol as random effect and did an analysis of variance (ANOVA) test with the null model excluding HLA-matching status. In addition, we did this analysis separately in unique cells and cells belonging to clones (cells having the same TRA and TRB CDR3)

$$\begin{array}{c} \text{H1}:\text{TRS}∼\text{HLA}-\text{matching status}+\text{experimental protocol}\; \end{array}$$

H0:TRS∼experimental protocol
