# Supplementary material for: Defining the genetic determinants of CD8+ T cell receptor repertoire in the context of immune checkpoint blockade
Source: Sci Adv. 2025 Jul 25;11(30):eadu3461. doi: 10.1126/sciadv.adu3461 (PMC12292918; doi:10.1126/sciadv.adu3461)

Science Advances
AAAS

Supplementary Materials for

Defining the genetic determinants of CD8$^+$ T cell receptor repertoire in the context of immune checkpoint blockade

Esther S. Ng *et al.*

Corresponding author: Benjamin P. Fairfax, benjamin.fairfax@oncology.ox.ac.uk

Sci. Adv. **11**, eadu3461 (2025)
DOI: 10.1126/sciadv.adu3461

The PDF file includes:

Figs. S1 to S14

Other Supplementary Material for this manuscript includes the following:

Tables S1 to S9

Supplementary Figure 1. Heatmap of correlation of α chain and β chain usage

α chain usage for each individual was correlated with β chain usage (Pearson method), producing a matrix of intra chain correlations which was then clustered according to Euclidean distances. The highest correlation was observed between TRAV1-2 and TRBV6-4 chains, corresponding to the known high degree of pairing of these chains within MAIT cells.

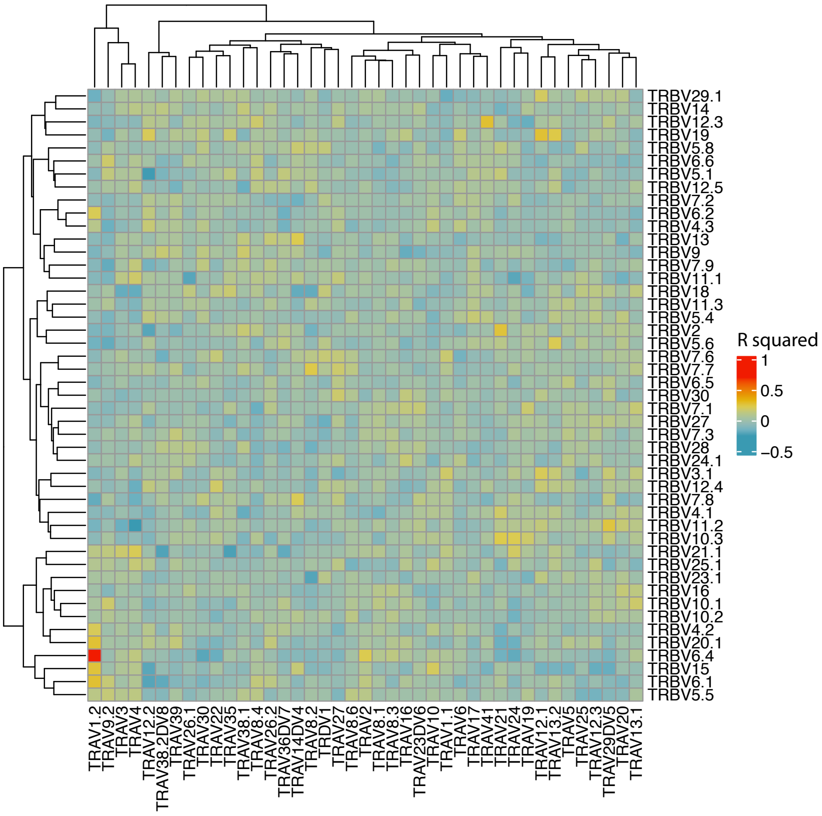

Supplementary Figure 2. Principal components of V-gene usage of pre- and post-ICB treatment samples. There is no clear separation between the two groups for (**A**) α chain (**B**) β chain.

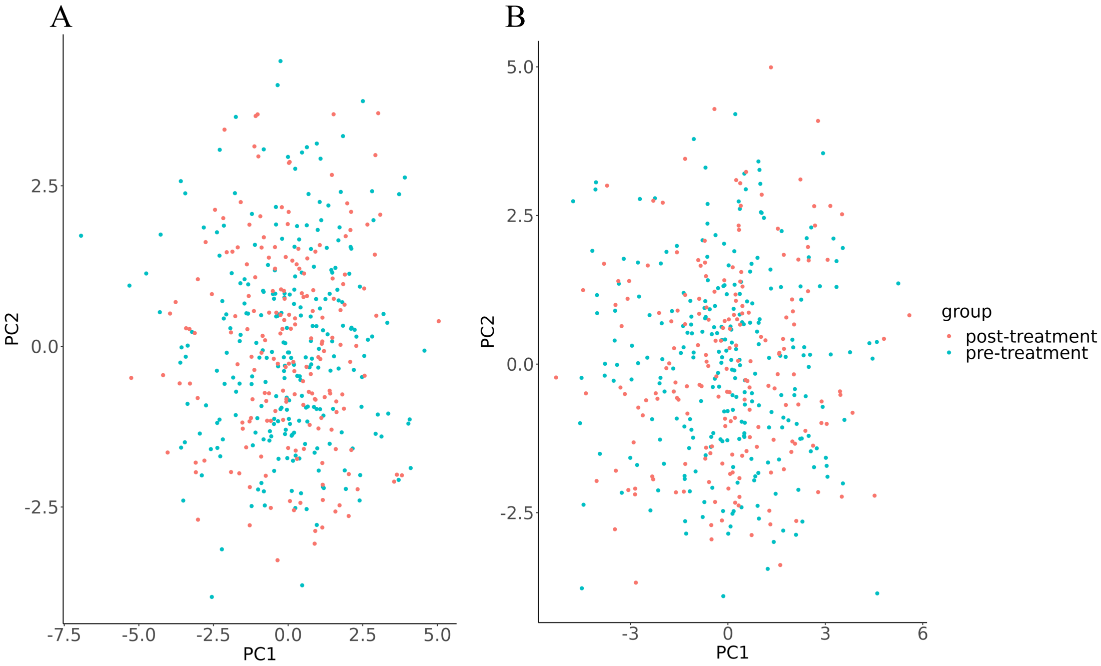

Supplementary Figure 3. Principal components of our cohort's genetic data plotted together with the 1000 Genomes project samples demonstrating that our samples are of European ancestry. Each of the dots represent one individual and are coloured by their population labels. AFR – African, AMR – Ad Mixed American, EAS – East Asian, EUR – European, SAS – South Asian.

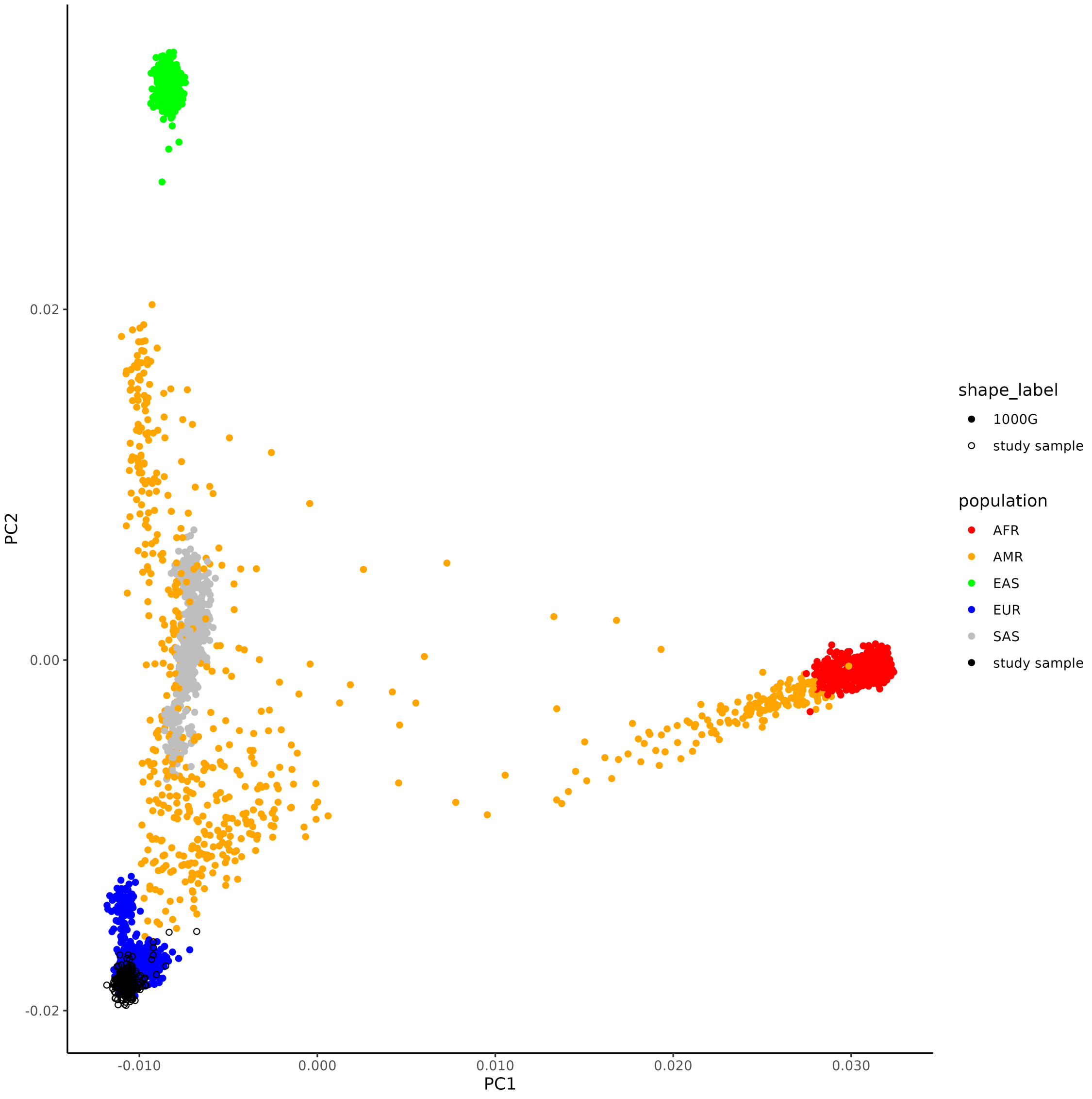

Supplementary Figure 4. Principal components of V-gene usage for TCR (**A**) α chain (**B**) β chain. Each point represents an individual and points are coloured by age, cancer type and gender from top to bottom respectively.

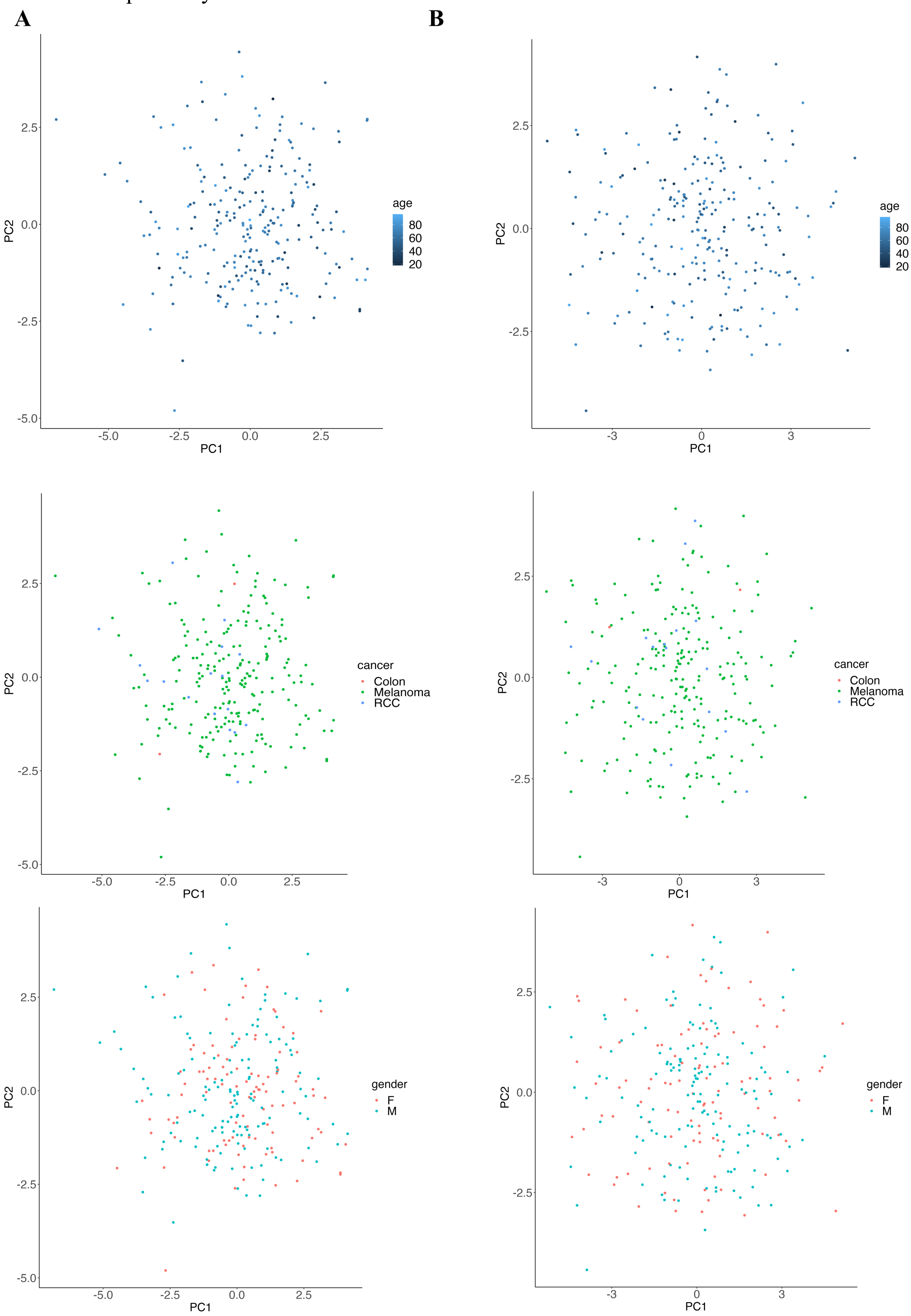

Supplementary Figure 5. Permutation analysis. To account for multiple testing and correlation between V-gene usage, we permuted the phenotype dataset 1000 times, preserving the V-genes that each individual had but reshuffling the samples. Plots are histograms of the permutation p-values for (**A**) α- and (**B**) β-chain V-gene usage GWAS (**C**) α- and (**D**) β chain MHC-wide association study. The 5% significance threshold is indicated by a red line.

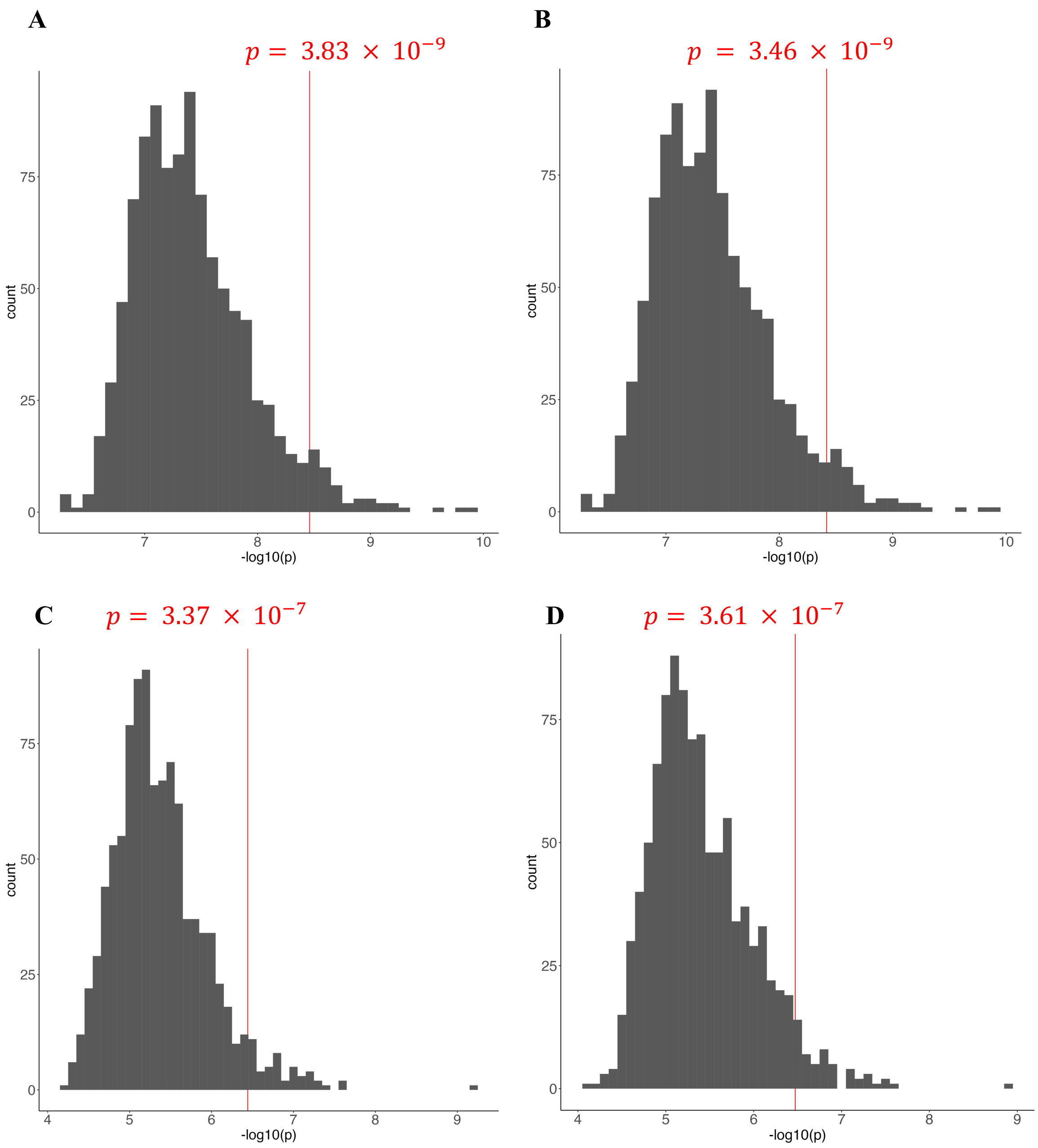

Supplementary Figure 6. Individual locus plots of V-gene HLA associations which pass significance threshold determined by permutation tests. Classical HLA genes are annotated in different colours annotated in the legend.

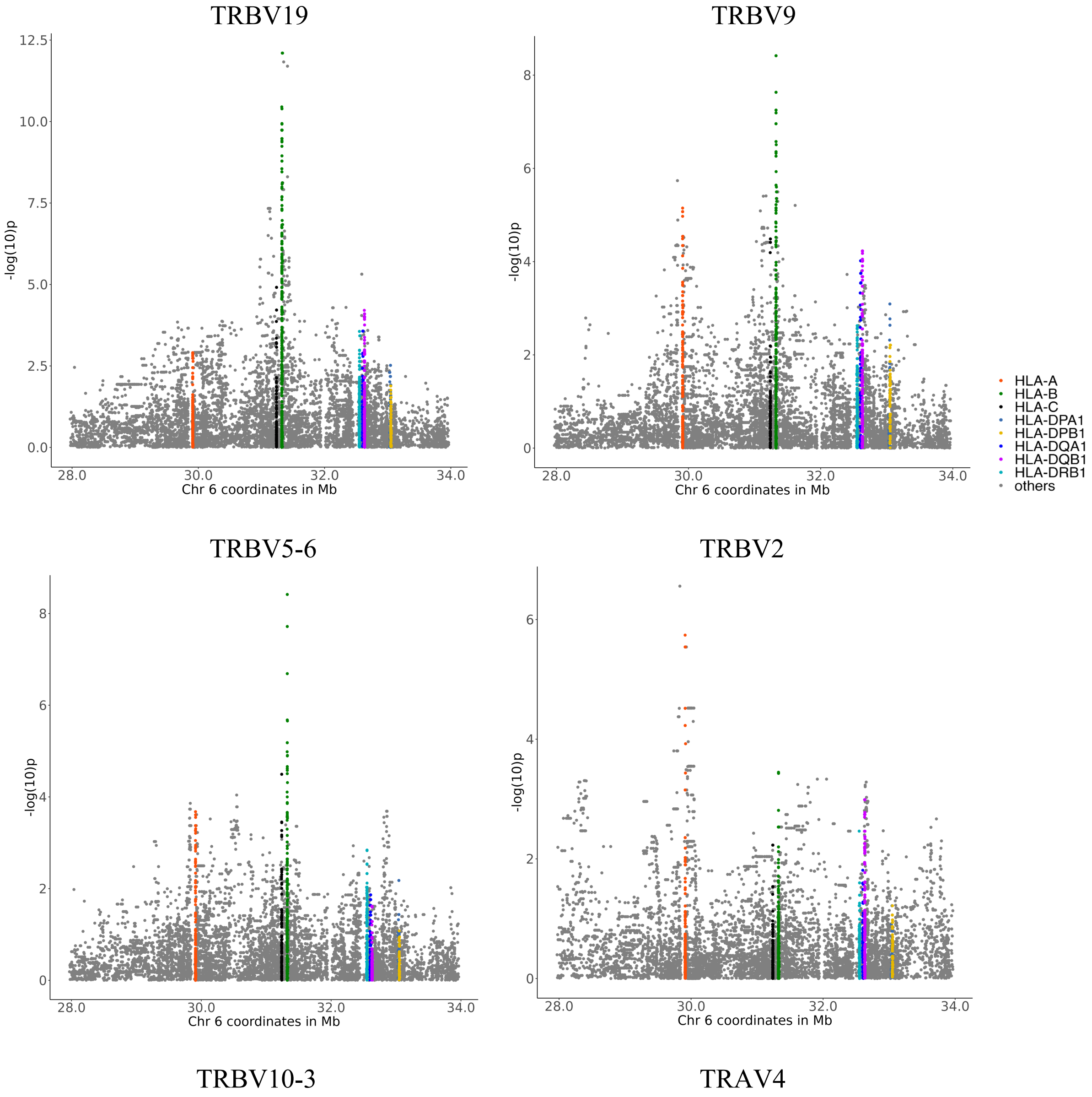

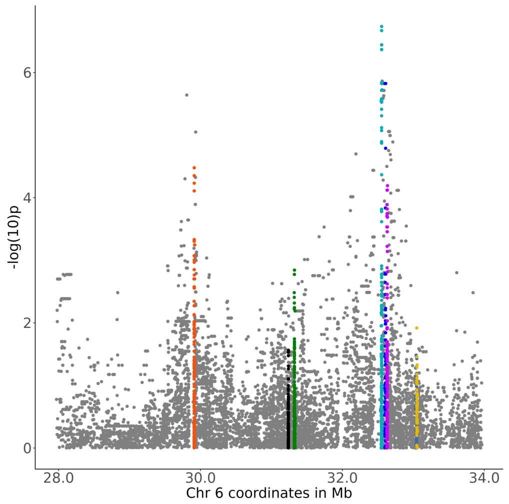

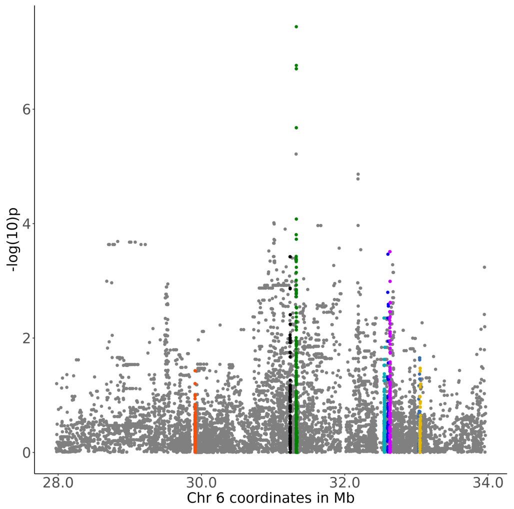

TRAV12-2

TRAV3

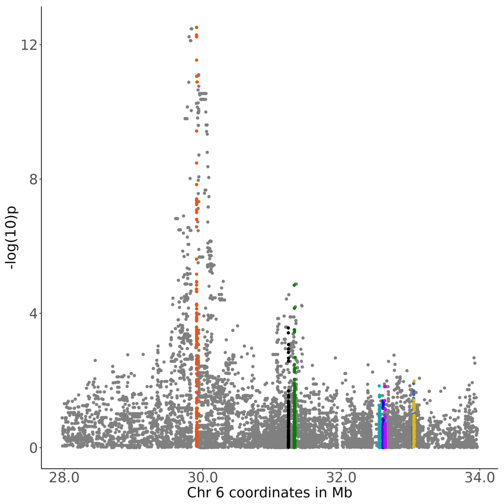

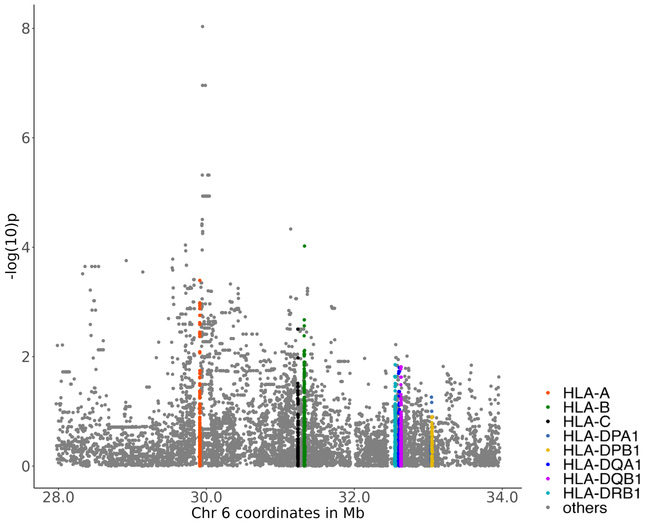

TRAV9-2

TRAV27

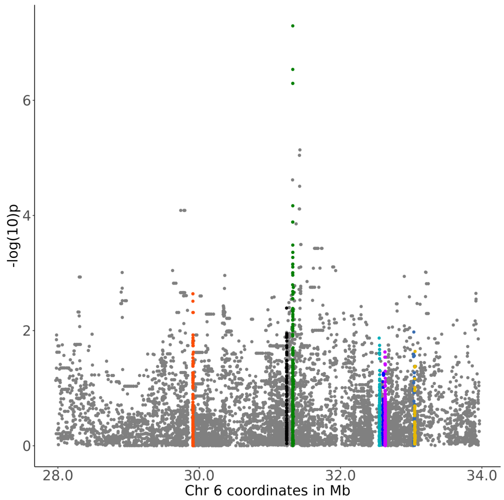

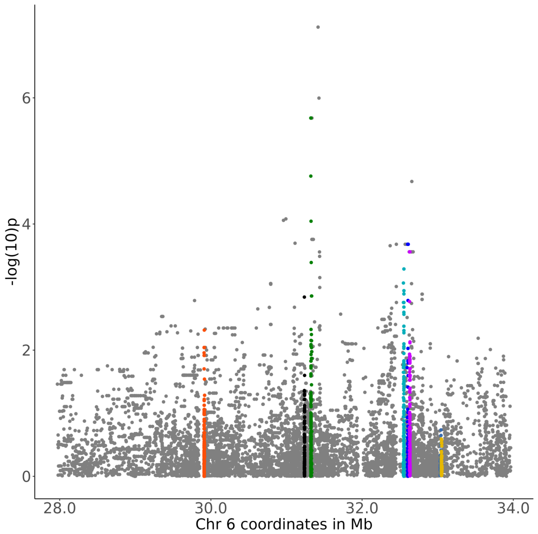

Supplementary Figure 7. Grid plots summarising the relationship between V-gene usage and 4-digit HLA. (**A**) α chain (**B**) β chain. Each square represents the p-value of the top independent signal for each V-gene.

A

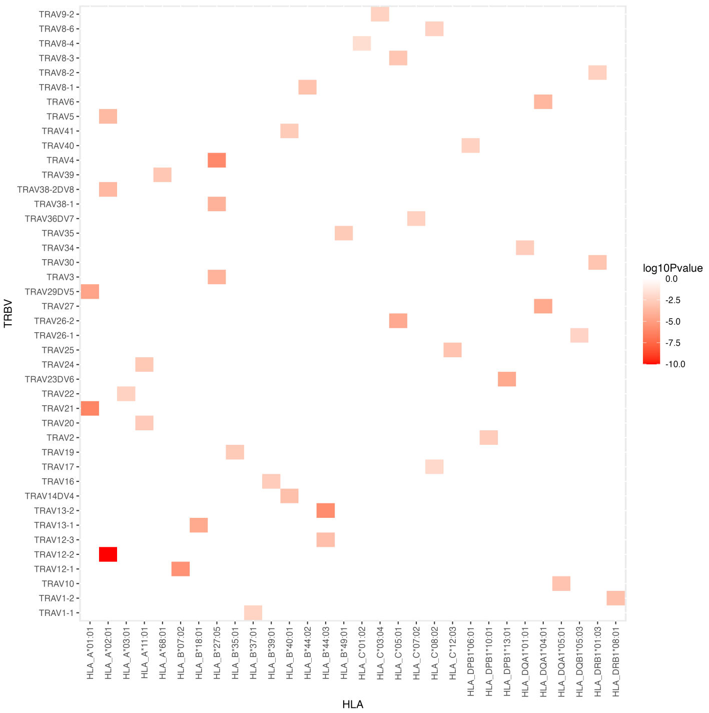

B

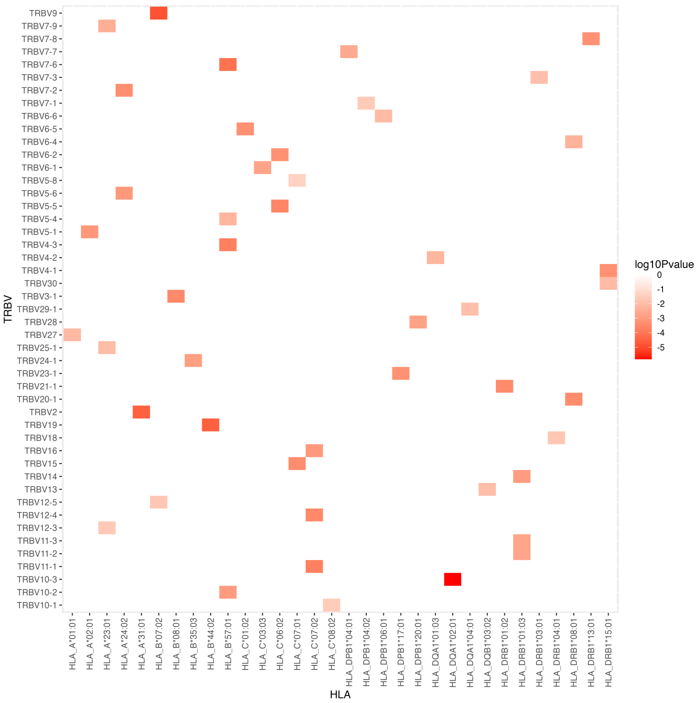

Supplementary Figure 8. Variance explained by each of the V-genes in the linear regression model against HLA for (**A**) α chain (**B**) β chain.

A

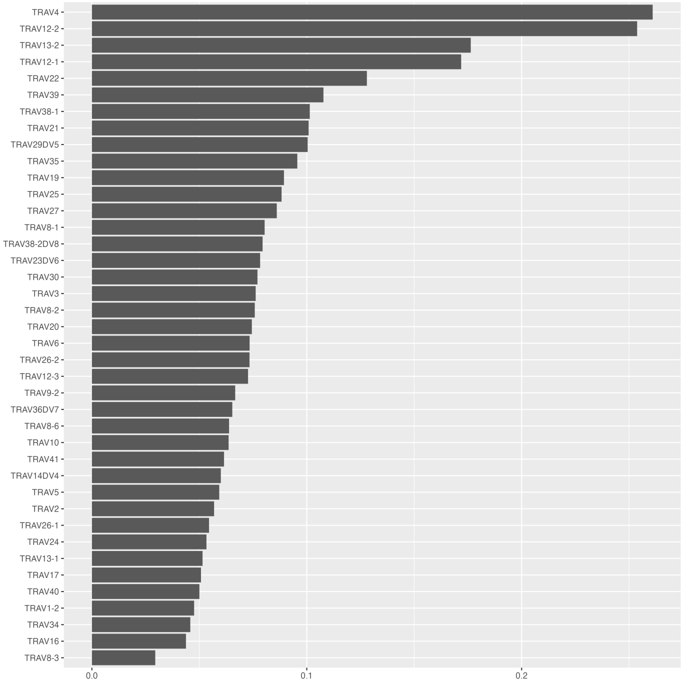

B

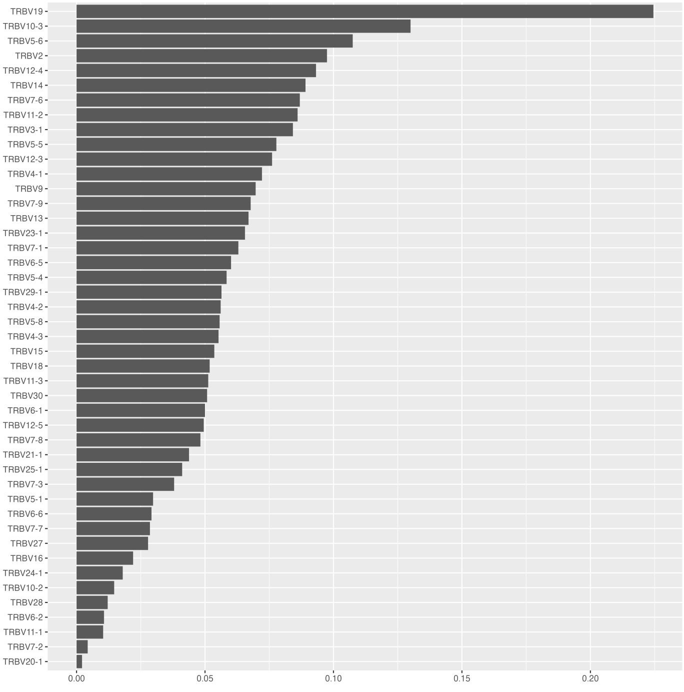

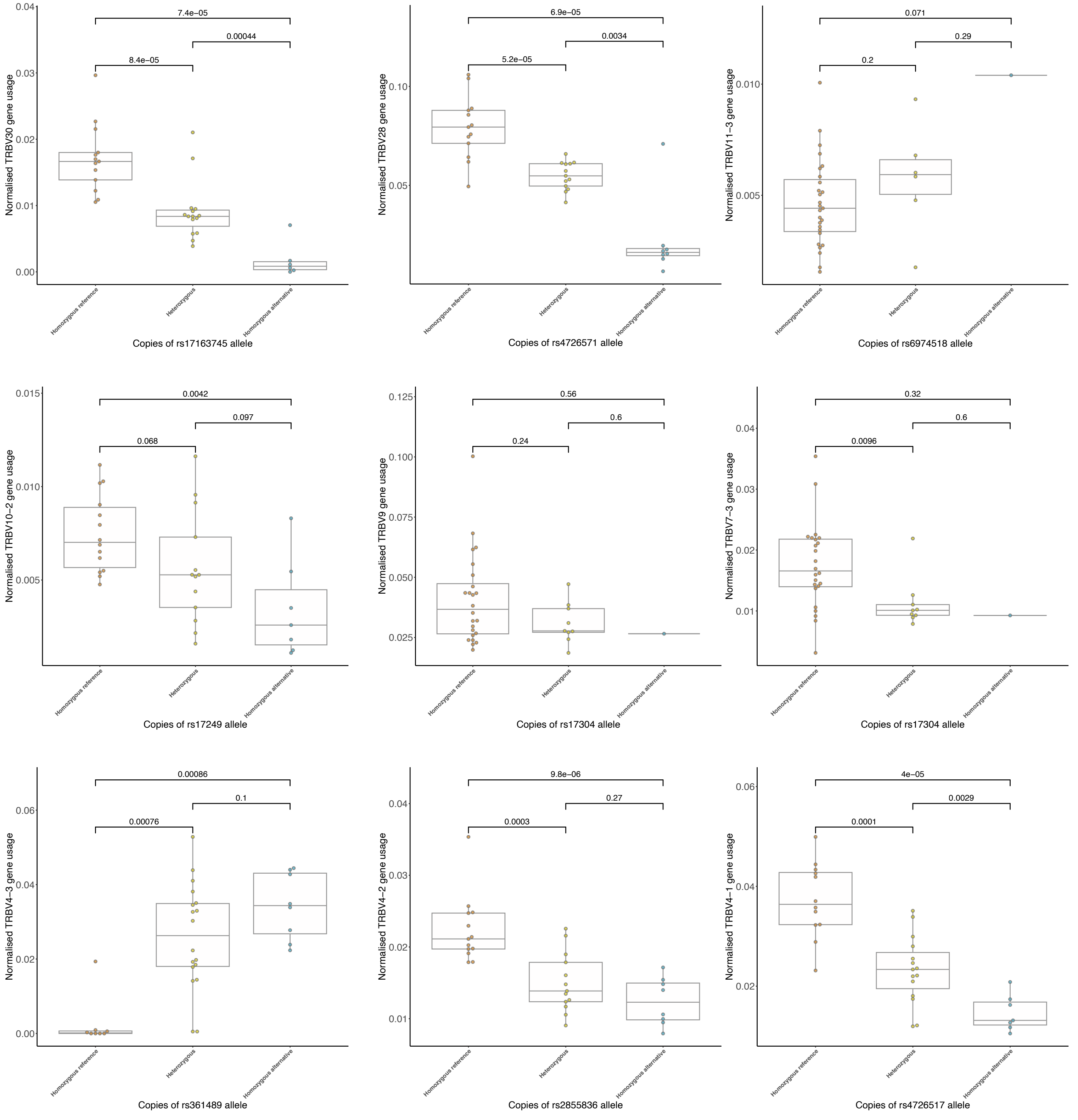

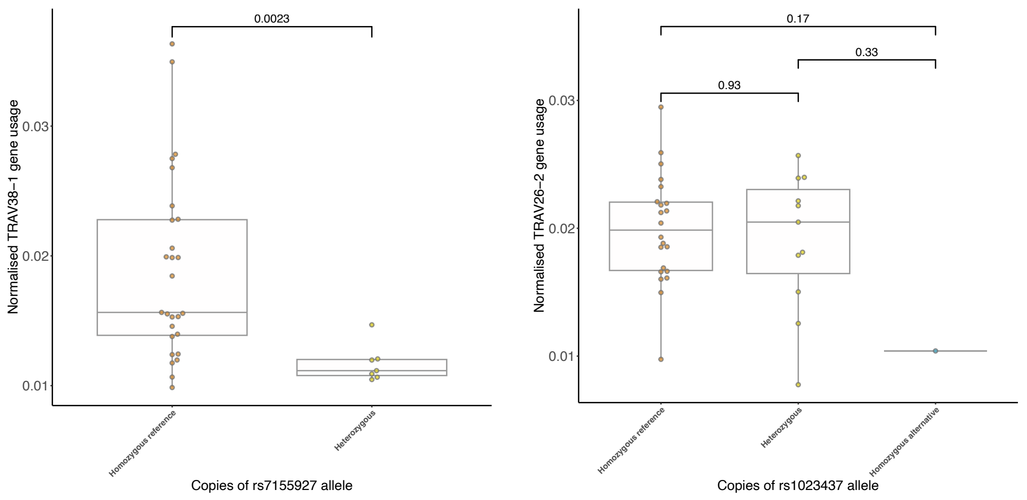

Supplementary Figure 9
Replication Cohort
TCR were mapped from baseline CD8+ T cell samples from 34 patients receiving ICB for cancer. We explored the association of all 11 primary GWAS observations in this smaller cohort, with 10/11 showing consistent direction of effect, and 9/11 statistical significance ($P<0.05$, Wilcoxon rank-sum tests)

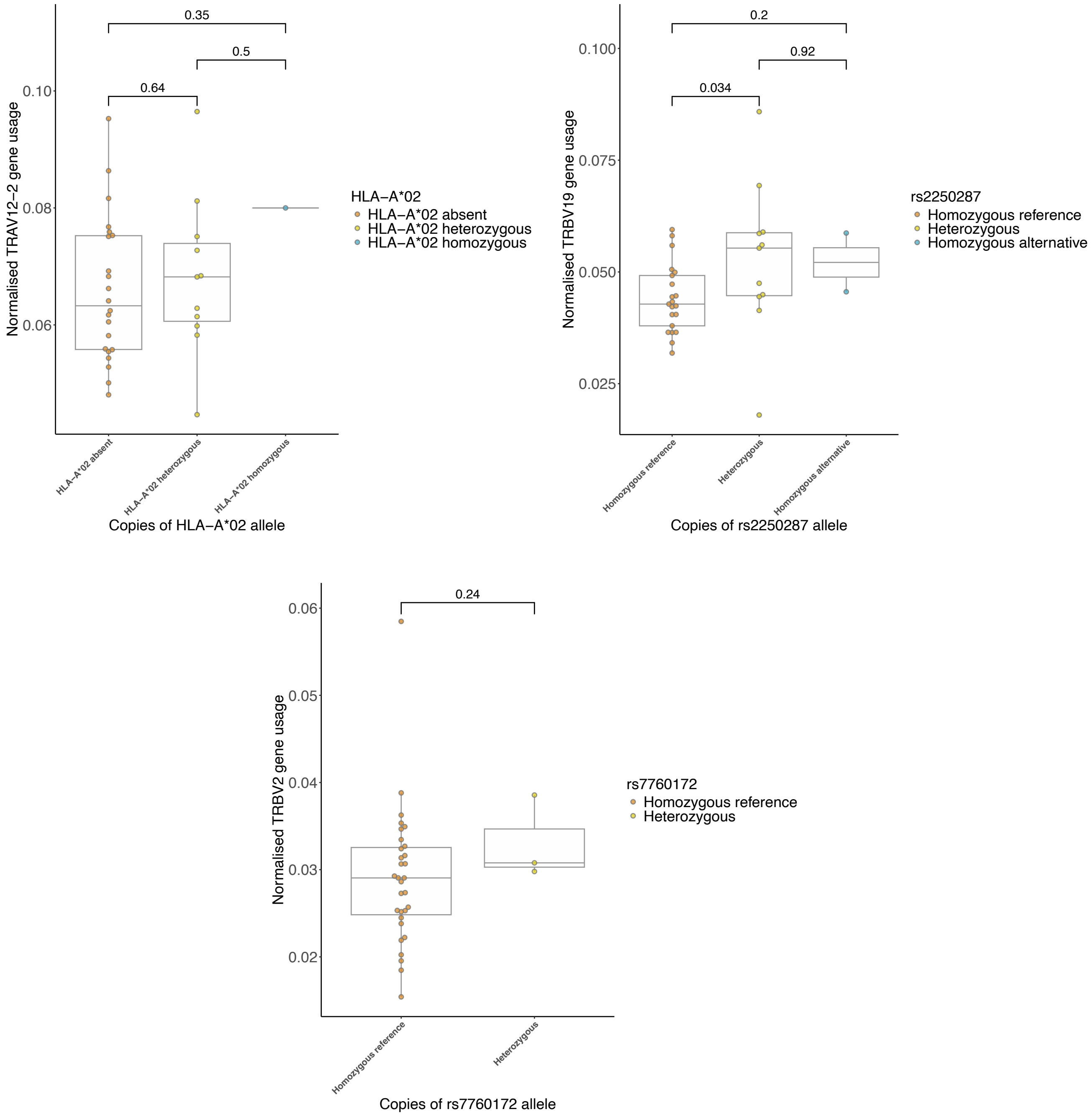

Supplementary Figure 10 Replication Cohort - *trans associations*
TCR were mapped from baseline CD8+ T cell samples from 34 patients receiving ICB for cancer. We explored the association of 5 of *trans* observations in this smaller cohort, with 3/5 showing consistent direction of effect despite low power ($P<0.05$, Wilcoxon rank-sum tests)

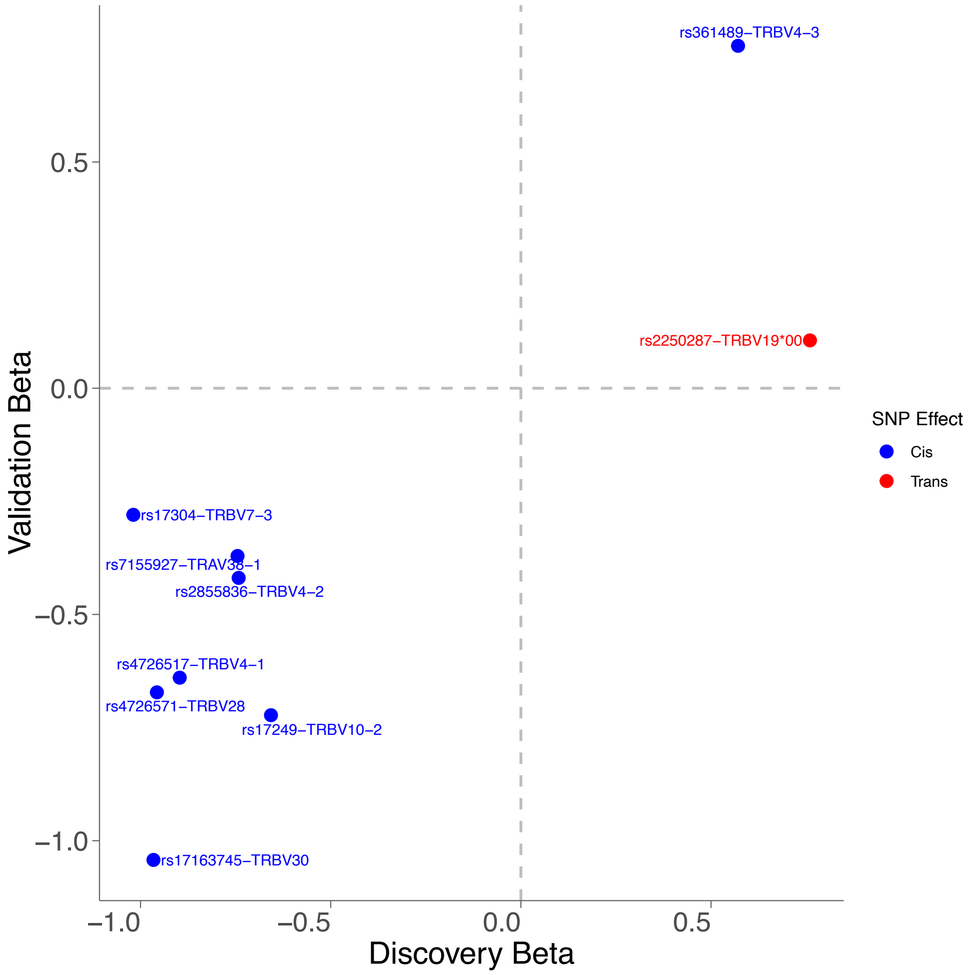

Supplementary Figure 11 Primary versus Replication Cohort Beta Values

The comparative beta values from primary and replication datasets for significantly replicated associations in the secondary cohort.

Supplementary Figure 12. Grid plots summarising the relationship between V-gene usage and amino acid positions in (**A**) α chain (**B**) β chain. The x-axis represents HLA and amino acid position eg. A105 is the 105th amino acid position in HLA. Each square represents the p-value of the top independent signal for each TRV gene

A

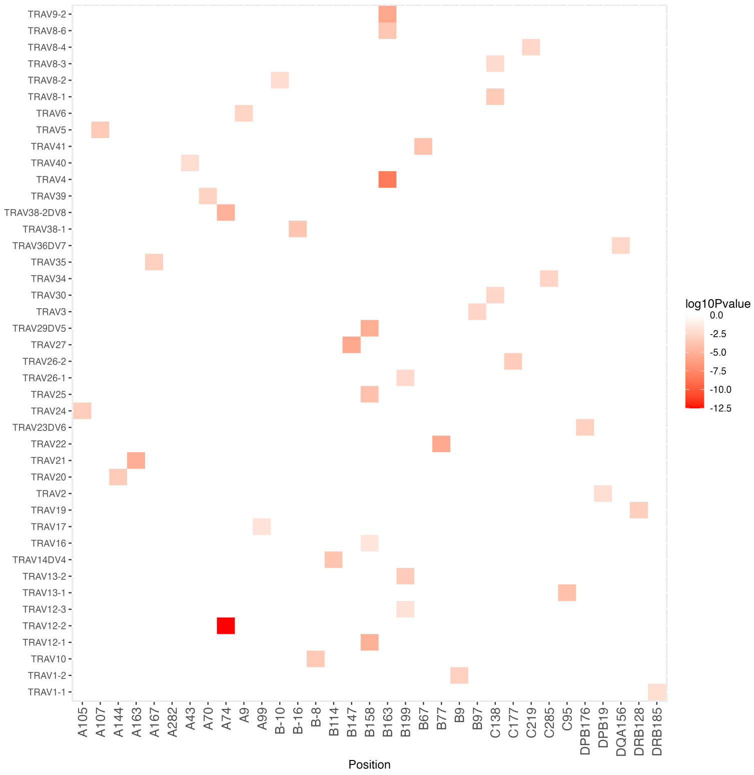

B

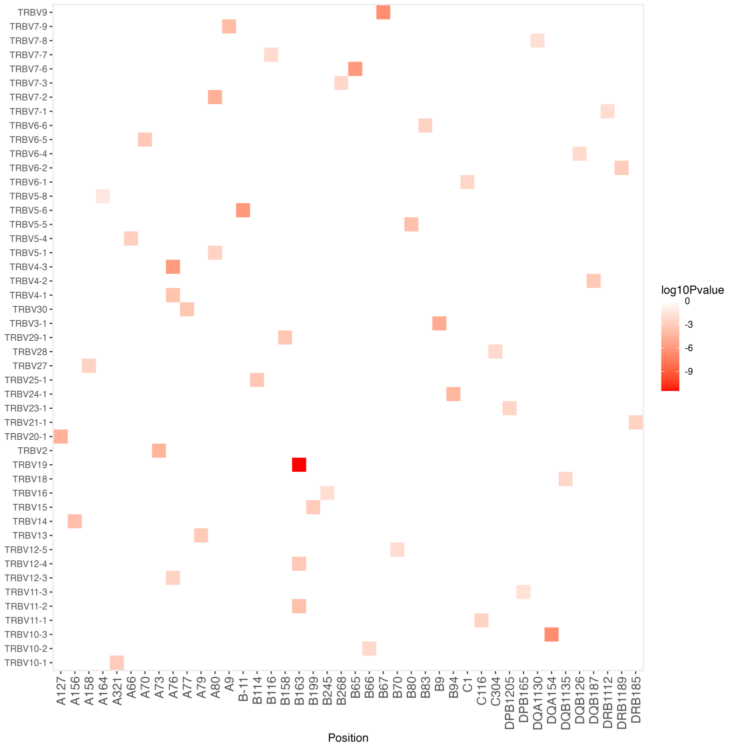

Supplementary Figure 13. Association between TCR CDR3 K-mer usage and germline genetic variation. Locus plot of association between variants in the MHC region and (**A**) TGDSNQP (**B**) TSGDYNE, both of which are on the TCR β chain.

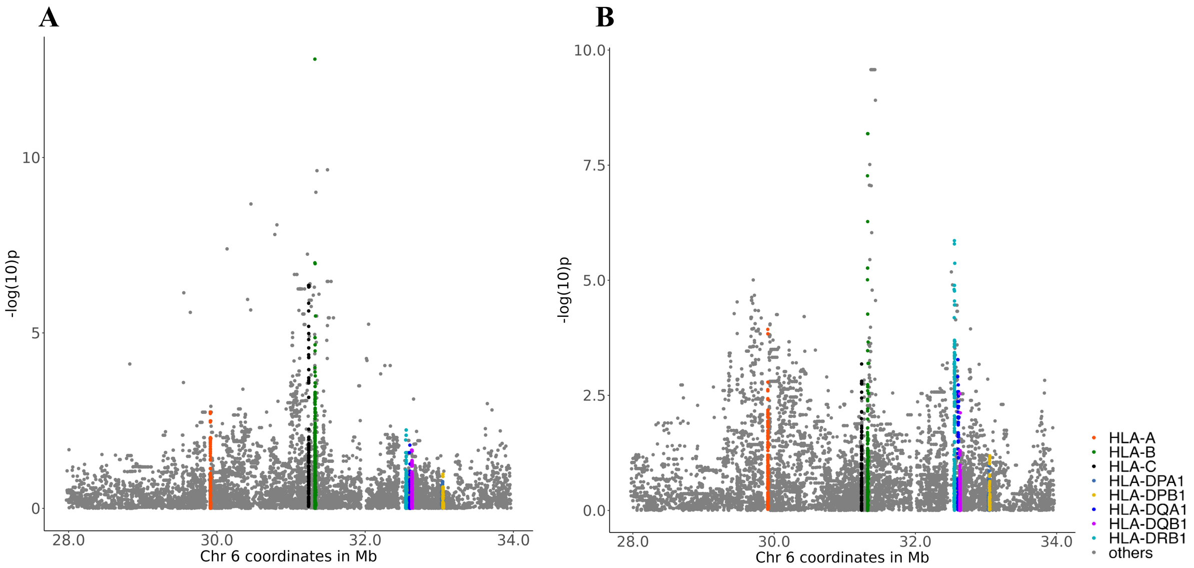

Supplementary Figure 14. Survival analysis of (**A**) all patients receiving ICB for metastatic disease for which we had pre-treatment samples demonstrated that patients carrying HLA-matched clones prior to treatment had improved overall survival. (**B**) patients receiving ICB for metastatic melanoma showed that carriage of HLA-matched clones either before or after treatment improved survival

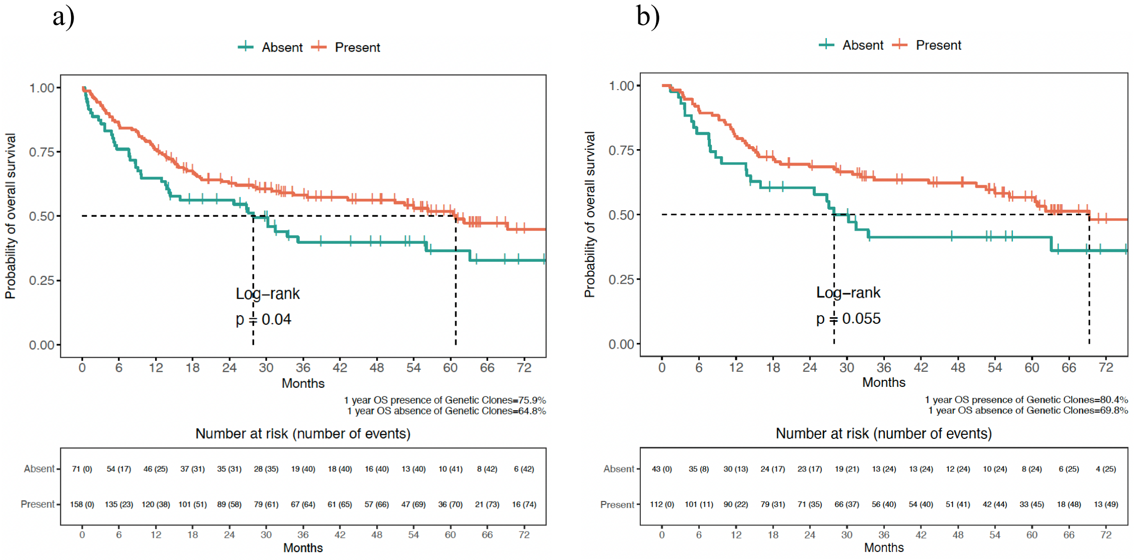

Supplement: Supplementary file 1 — Figs. S1 to S14 [file sciadv.adu3461_sm.pdf]
